# The planktonic microbiome of the Great Barrier Reef

**DOI:** 10.1038/s41586-026-10778-z

**Published:** 2026-07-22

**Authors:** Steven Robbins, Marko Terzin, Katherine Dougan, Julian Zaugg, Sara C. Bell, Patrick W. Laffy, J. Pamela Engelberts, Kim-Anh Lê Cao, Renee K. Gruber, Nicole S. Webster, David G. Bourne, Philip Hugenholtz, Yun Kit Yeoh

**Affiliations:** 1 Australian Centre for Ecogenomics, School of Chemistry and Molecular Biosciences, The University of Queensland, St Lucia, Queensland Australia; 2 Australian Institute of Marine Science, Townsville, Queensland Australia; 3 AIMS@JCU, Research Division, James Cook University, Townsville, Queensland Australia; 4 College of Science and Engineering, James Cook University, Townsville, Queensland Australia; 5 Melbourne Integrative Genomics, School of Mathematics and Statistics, University of Melbourne, Parkville, Victoria Australia; 6 University of Tasmania, Battery Point, Tasmania Australia

**Keywords:** Water microbiology, Metagenomics, Microbial ecology, Microbiome

## Abstract

Large genome databases have markedly improved our understanding of marine microorganisms^1–5^. Although these resources have focused on prokaryotes, genomes from many dominant marine lineages, such as *Pelagibacter* and *Prochlorococcus*, are conspicuously underrepresented. Here we present the Great Barrier Reef Microbial Genomes Database (GBR-MGD), comprising 5,283 prokaryotic genomes obtained from Great Barrier Reef seawater samples using Nanopore and Illumina sequencing, including a collection of high-quality genomes of underrepresented groups. We show that standard short-read assemblies miss these populations owing to a combination of strain heterogeneity and low-GC-percentage sequencing bias. The GBR-MGD also comprises 20 chromosome-level picoeukaryote and 808,585 viral genomes, including a newly described clade of marine *Crassvirales*. We demonstrate the utility of the GBR-MGD to identify indicator taxa that can reliably predict the effects of reef management practices, such as the establishment of marine protected zones.

## Abstract (web summary)

The Great Barrier Reef Microbial Genomes Database compiles prokaryotic, viral and eukaryotic genomes from seawater collected from the Great Barrier Reef, providing a rich resource for the study of marine microbial communities and ecosystem research and management.

## Main

Microorganisms dominate life in the oceans^6^, forming the foundation of marine food webs^7^ and driving global biogeochemical cycles^8^ through the metabolic interactions of bacterial, archaeal, viral and microbial eukaryotic communities. Understanding how these communities function, interact and change over time is critical for predicting ecological resilience, especially as oceans come under increasing pressure from climate change^9^. The overwhelming majority of microbial species found in marine ecosystems have yet to be cultured; however, the establishment of large-scale databases of prokaryote genomes via metagenomic sequencing has markedly improved our understanding of the identity, functional potential and distribution of marine microorganisms^1–5^. Among the prokaryotic inventories, however, many of the dominant species are conspicuously underrepresented, as evidenced by flow cytometry cell counts and metagenomic read mapping, including *Pelagibacter*, *Prochlorococcus*, SAR86 and others^5,10^. Further, the viral and eukaryote components of metagenomic surveys are less often included, with a few notable exceptions^11–17^. The oceans around Australia are also mostly absent in global marine genome surveys^4,18^, as are its coral reefs^19,20^, which are hotspots for biodiversity^9^. To address these gaps, we provide a detailed assessment of the pelagic microbiome of the Great Barrier Reef (GBR), the world’s largest coral reef, using hybrid long-read and short-read DNA sequencing to construct a holistic database of bacteria, archaea, eukaryotes, viruses and plasmids, collectively termed the Great Barrier Reef Microbial Genomes Database (GBR-MGD).

We show that several prokaryote taxa are recalcitrant to recovery with Illumina short reads alone, owing to a combination of high strain heterogeneity and a bias against low-GC taxa, which can be rectified by inclusion of Nanopore long-read sequencing. This technology also enabled us to assemble *Crassvirales* genomes that form a distinct group within the order, as well as chromosome-level picoeukaryotic metagenome-assembled genomes (eMAGs) from multiple *Bathycoccus* structural variants and the as yet undescribed *Ostreococcus* clade B, often the most common picoeukaryote found on coral reefs. We were able to leverage the GBR-MGD prokaryote metagenome-assembled genomes (pMAGs) and machine learning techniques to identify reproducible shifts in community structure that reflect the GBR Marine Park Authority’s management plan designating reefs as open or closed to fishing. To our knowledge, this is the first demonstration of a large-scale ecosystem-wide effect of fisheries management on microbial communities of the surrounding seawater. This study marks a substantial advance in our ability to recover complete and near-complete prokaryotic, eukaryotic and viral genomes from seawater samples, enabling marine ecosystem research and management.

## Benchmarking hybrid metagenome assemblies

**Fig. 1:**
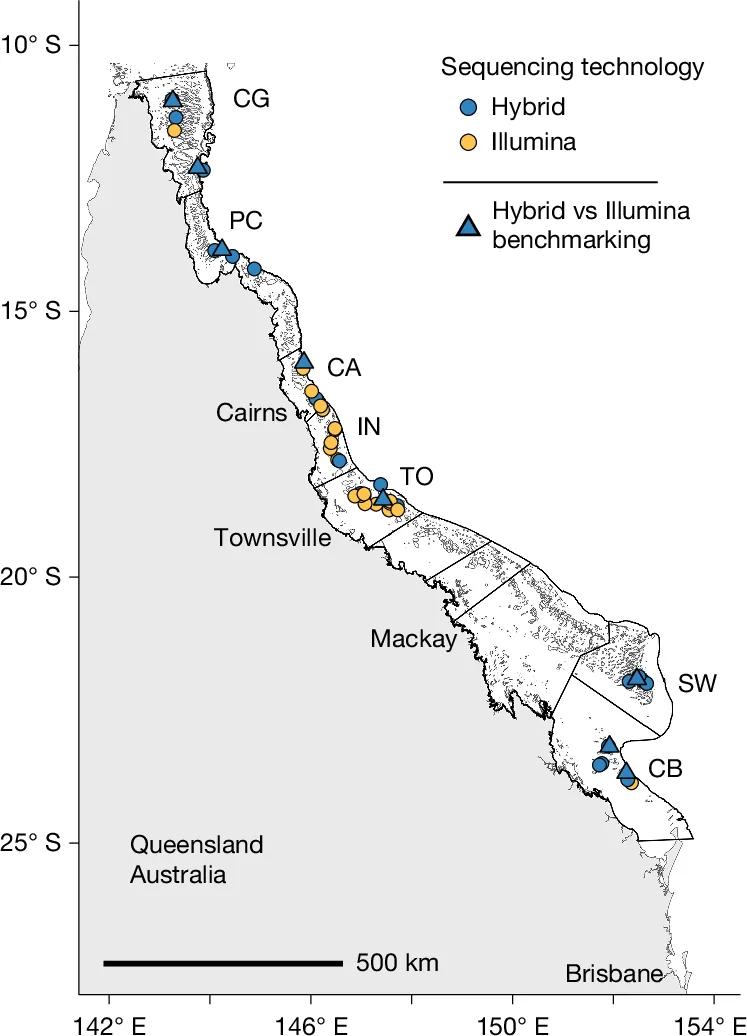
Location of the 48 sites sampled across the GBR. Sampling was conducted alongside reef surveys performed by the AIMS LTMP. At each site, 4 × 5 l replicate seawater samples were collected for DNA extraction. Sites with Nanopore and Illumina sequence data (hybrid) are indicated with blue circles or triangles while sites with Illumina data only are indicated in yellow. Blue triangles represent the eight sites used for benchmarking Nanopore and Illumina hybrid versus Illumina-only assemblies. Sector names are indicated next to their locations. CA, Cairns; CB, Capricorn Bunker; CG, Cape Grenville; IN, Innisfail; PC, Princess Charlotte Bay; SW, Swain; TO, Townsville.

The absence of representative microbial genomic surveys of the GBR hampers our ability to understand how reef microbial communities are changing, especially in response to anthropogenic stressors such as climate change. To generate a comprehensive database of GBR marine microorganisms, seawater samples were collected from 48 sites spanning a north-to-south transect of the GBR (Fig. 1) for metagenomic sequencing (Supplementary Tables 1–3). To enhance metagenome-assembled genome (MAG) recovery, we used a hybrid assembly of long and short reads, which has been shown to substantially improve the quality of MAGs compared to short-read assemblies^21^.

**Fig. 2:**
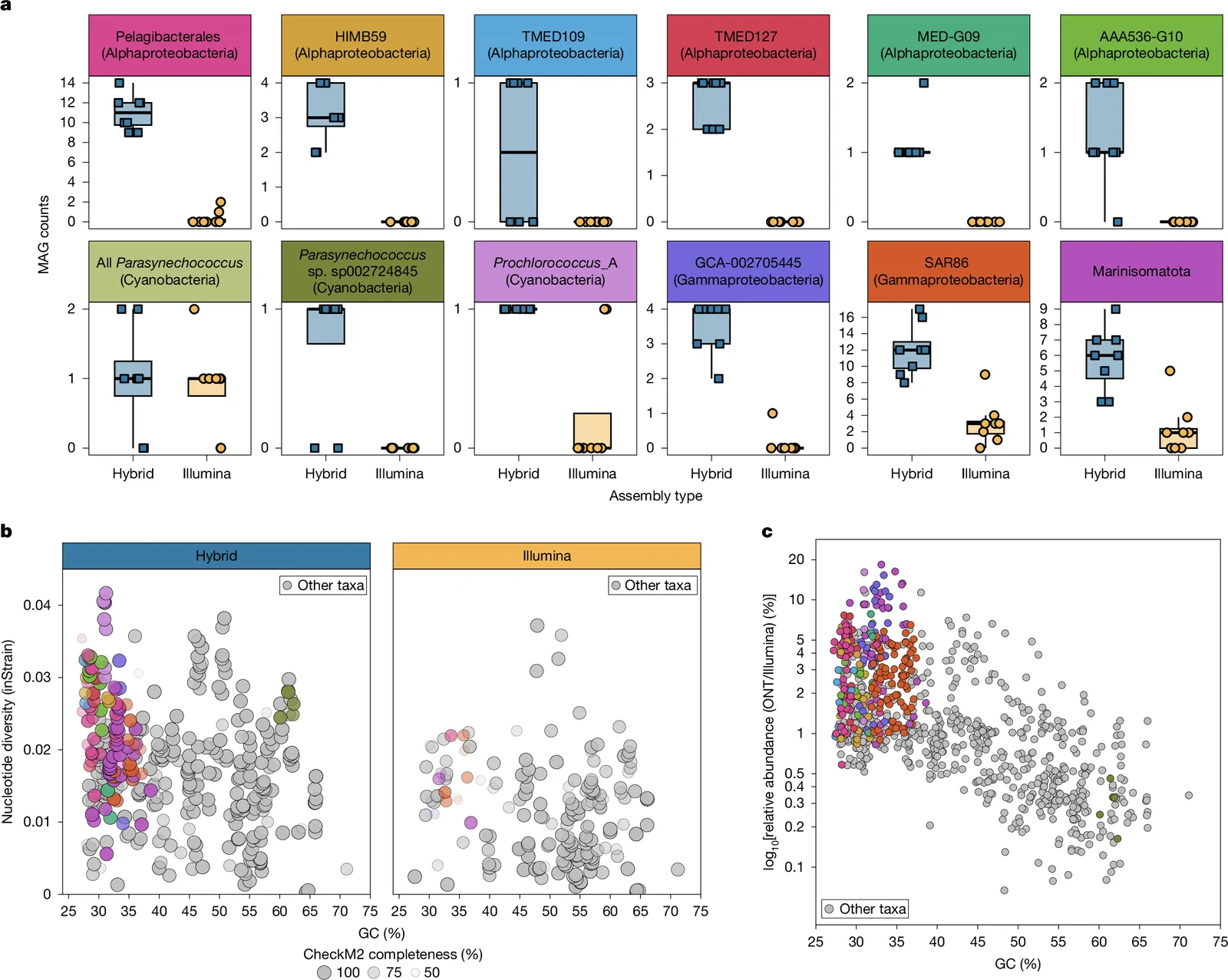
Plots illustrating recovery and sequencing bias between Illumina-only and Nanopore hybrid pMAGs. **a**, Box-and-whisker plots showing number of recovered pMAGs per sample for taxa that are underrepresented in Illumina-only assemblies (*n* = 8 samples from different sites used for comparative Illumina-only versus hybrid assembly benchmarking). Centre lines in the boxes represent median values, top and bottom box boundaries represent top and bottom quartiles, respectively, whiskers extend to 1.5× interquartile range, and minima and maxima are presented as individual data points. **b**, Plots of nucleotide diversity (a measure of genetic diversity within microbial populations implemented in inStrain) and average GC percentage for hybrid (left; *n* = 382) and Illumina-only (right; *n* = 179) benchmark pMAGs after species-level dereplication (95% ANI), highlighting taxa that are challenging to recover using short reads in **a**. Circle opacity and size are scaled according to CheckM2 completeness. **c**, Plot showing Nanopore overrepresentation as a function of average MAG GC percentage across all benchmark hybrid (*n* = 987) and Illumina-only (*n* = 516) pMAGs, where overrepresentation is defined as the ratio of pMAG relative abundance in Nanopore versus Illumina reads. All MAGs have CheckM2 quality scores >50 and contamination <10%. Circle colours in panels **b** and **c** correspond to taxa colours in **a**.

**Extended Data Fig. 1:**
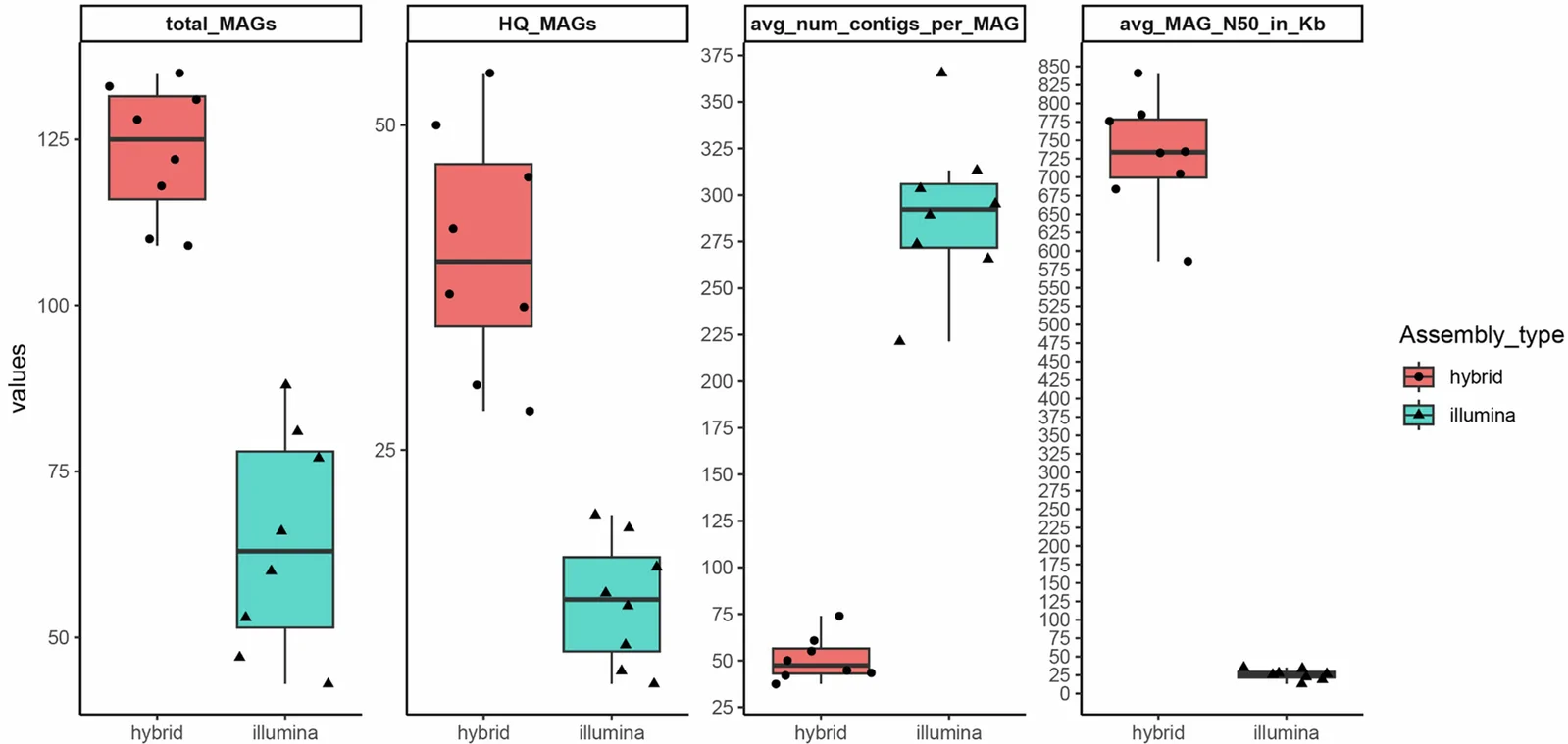
Metagenome-assembled genome (MAG) quality statistics for Illumina-only and hybrid metagenome assembly benchmarking. Box and whisker plots showing the number of MAGs recovered with quality >50 (%completeness – 3x %contamination) from Illumina-only and Nanopore Hybrid metagenome assemblies (n = 8 samples from eight different reefs used for comparative Illumina-only vs. hybrid assembly benchmarking and MAG binning), number of high-quality MAGs recovered, average number of contigs per MAG, and the average MAG N50. Centre lines of the box plots represent median values, upper and lower box boundaries represent upper and lower quartiles, respectively, whiskers extend to 1.5x interquartile range, and value minima and maxima are represented by data points.

We hypothesized that common but difficult-to-recover marine taxa such as *Pelagibacter* could be obtained using Nanopore long-read sequencing to span strain-variable regions based on the expectation of high strain heterogeneity^22^. To test this hypothesis, we selected eight seawater samples for Illumina-only and hybrid (Illumina plus Nanopore) assemblies and compared the recovery of medium- to high-quality pMAGs with a focus on lineages previously found to be difficult to recover as MAGs. The total number of base pairs in both Illumina-only and hybrid assemblies was standardized to 30 Gbp to remove sequencing depth as a factor. Contiguity of the hybrid-assembled pMAGs was 29-fold higher than that of the Illumina-only pMAGs (N50 of 730 versus 25 kb), exceeding the genome length of some marine taxa (for example, Nanoarchaeota^23^; Fig. 2a, Supplementary Tables 4 and 5 and Extended Data Fig. 1). Commensurately, the average number of medium to high-quality pMAGs per sample increased about twofold in hybrid assemblies (64 to 123) with the increase even more apparent for high-quality pMAGs (13 to 40; Fig. 2a and Extended Data Fig. 1).

The increased number of hybrid pMAGs included lineages that were previously identified as difficult to recover with short-read-only sequencing, including Pelagibacterales, *Parasynechococcus*, *Prochlorococcus* and SAR86, along with other lineages that were not previously recognized as difficult to recover. For example, pMAGs from the Alphaproteobacteria class HIMB59, TMED109, TMED127 and MED-G09, the family AAA536-G10 within the order Puniceispirillales (formerly SAR116), and the Gammaproteobacteria order GCA-002705445, were not recovered from Illumina-only assemblies but were consistently recovered in hybrid assemblies (Fig. 2a). Notably, genomes belonging to the order Pelagibacterales and phylum Marinisomatota (formerly Marinimicrobia and SAR406) were recovered infrequently in Illumina-only assemblies, whereas 5 to 17 pMAGs were recovered per sample from the hybrid assemblies (Fig. 2a), resulting in substantial missed diversity using short read only assemblies—25 versus 2 different species across the 8 samples for Pelagibacterales and 13 versus 7 species in Marinisomatota (Supplementary Tables 4 and 5). Within the Cyanobacteriota, *Prochlorococcus* pMAGs were generally not recovered from Illumina-only assemblies but were recovered from all hybrid assemblies. Notably, whereas *Parasynechococcus* (formerly *Synechococcus*_E) pMAGs could be recovered from both short-read and hybrid assemblies, only the hybrid assemblies recovered representatives of *Parasynechococcus* species sp002724845 (Fig. 2a), the most dominant prokaryote lineage in GBR seawater, averaging 8.9 ± 5.9% relative abundance (maximum 35.9%) across the complete GBR-MGD dataset. By comparison, the next most abundant *Parasynechococcus* species averaged only 0.2% (Supplementary Table 6).

We next investigated whether strain heterogeneity was the driving factor behind the improved recovery of pMAGs using Nanopore hybrid assemblies. One way to assess strain heterogeneity is to quantify single nucleotide polymorphism frequencies, and indeed, a high proportion of hybrid pMAGs from difficult-to-recover taxa represent populations with high nucleotide diversity (>0.02; Fig. 2b), a measure of genetic diversity within microbial populations implemented in inStrain^24^. By contrast, Illumina-only pMAGs rarely have nucleotide diversity values greater than 0.02, indicating a threshold above which short-read pMAG recovery is severely affected (Fig. 2b). However, several difficult-to-recover taxa have sufficiently low strain heterogeneity (0.005 to 0.02 inStrain nucleotide diversity; Fig. 2b) to be recovered by Illumina-only assembly, suggesting a secondary reason for improved pMAG recovery in hybrid assemblies. By mapping both the Illumina and Nanopore reads used in hybrid assemblies back to their respective pMAGs, we found that difficult-to-recover taxa were consistently under-represented in Illumina datasets (up to 18× less than in Nanopore datasets; Fig. 2c). Plotting GC content and fold Nanopore overrepresentation for all pMAGs, defined as the relative abundance in Nanopore reads divided by the relative abundance in Illumina reads, we found that low-GC pMAGs were better represented in Nanopore than Illumina data (Fig. 2c). For example, Marinisomatota pMAGs are up to 18× less abundant (average 6×) in Illumina reads than Nanopore, *Prochlorococcus* are up to 16× less abundant (average 7×), Gammaproteobacteria GCA-002705445 are up to 15× (average 6×) less abundant, and *Pelagibacter* are up to 7× (average 3×) less abundant. In addition to high nucleotide diversity, this would account for the limitations of Illumina-only assemblies in recovering pMAGs from difficult taxa, as all have GC contents below 40%. This is consistent with a known GC bias in polymerase-based sequencing platforms, including Illumina^25^. When considering both GC bias and strain diversity as factors, short-read MAG recovery appears to be difficult above 0.005 inStrain nucleotide diversity and less than 40% GC, as few pMAGs were recovered within this range and those that were showed lower completeness values (Fig. 2b). The problem reaches a critical threshold above 0.02 nucleotide diversity and <40% GC, where no pMAGs could be recovered from the short-read assemblies, despite many difficult taxa in this range being readily recovered in hybrid assemblies (56 hybrid versus 0 Illumina pMAGs) (Fig. 2b). In summary, we show that assemblies that incorporate long reads substantially enhance the recovery of dominant and ubiquitous marine lineages that are often missed by short-read-only assemblies, not only because long reads can overcome elevated strain heterogeneity but because of GC bias inherent to Illumina sequencing.

## Recovery of prokaryote MAGs

Having established that Nanopore sequencing substantially improves representation of cosmopolitan marine lineages, we extended our efforts to additional samples to produce the GBR-MGD. Hybrid assemblies from 27 of the 48 GBR seawater samples yielded 4,713 bacterial and archaeal pMAGs, including 1,505 high-quality (>90% completeness with <5% contamination) and 345 circular pMAGs assumed to be complete. We supplemented these data with Illumina-only assemblies from the remaining 21 sites resulting in an additional 570 pMAGs (85 in high quality), bringing the total number of bacterial and archaeal pMAGs to 5,283 in the final GBR-MGD database. Of the taxa identified to be recalcitrant to pMAG recovery, we obtained 319 Pelagibacterales pMAGs (22 high quality, 7 circular), 445 SAR86 (128 high quality, 35 circular), 222 Marinisomatota (70 high quality, 14 circular), 21 *Parasynechococcus* sp002724845 (1 high quality, 0 circular), 26 *Prochlorococcus* (8 high quality, 0 circular), 135 Gammaproteobacteria order GCA-002705445 (67 high quality, 34 circular) and 45 Alphaproteobacteria family AAA536-G10 (4 high quality, 0 circular). The recovery of circular pMAGs also enabled us to identify lineages for which CheckM version 1 (CheckM1) systematically underestimates completeness, as circular pMAGs should technically have a completeness value of 100% (the exception being multipartite genomes containing two or more replicons). Indeed, all circular genomes from the following taxa had completeness estimates below 85%: Gammaproteobacteria orders SAR86 and GCA-002705445, Bacteroidota genus MED-G13, Chloroflexota order UBA1151, Desulfobacterota class UBA1144, and the archaeal order Poseidonales (Supplementary Table 7). Completeness estimates were substantially higher for circular genomes using CheckM2, averaging 91% versus 79% for CheckM1, though groups GCA-002705445 and SAR86 are still underestimated using CheckM2 (average approximately 75% completeness) despite being circular. This underscores the need to use CheckM2 for completeness estimates of marine pMAGs to avoid discarding systematically underestimated genomes, which is likely to have contributed to underrepresentation of certain taxa.

**Extended Data Fig. 2:**
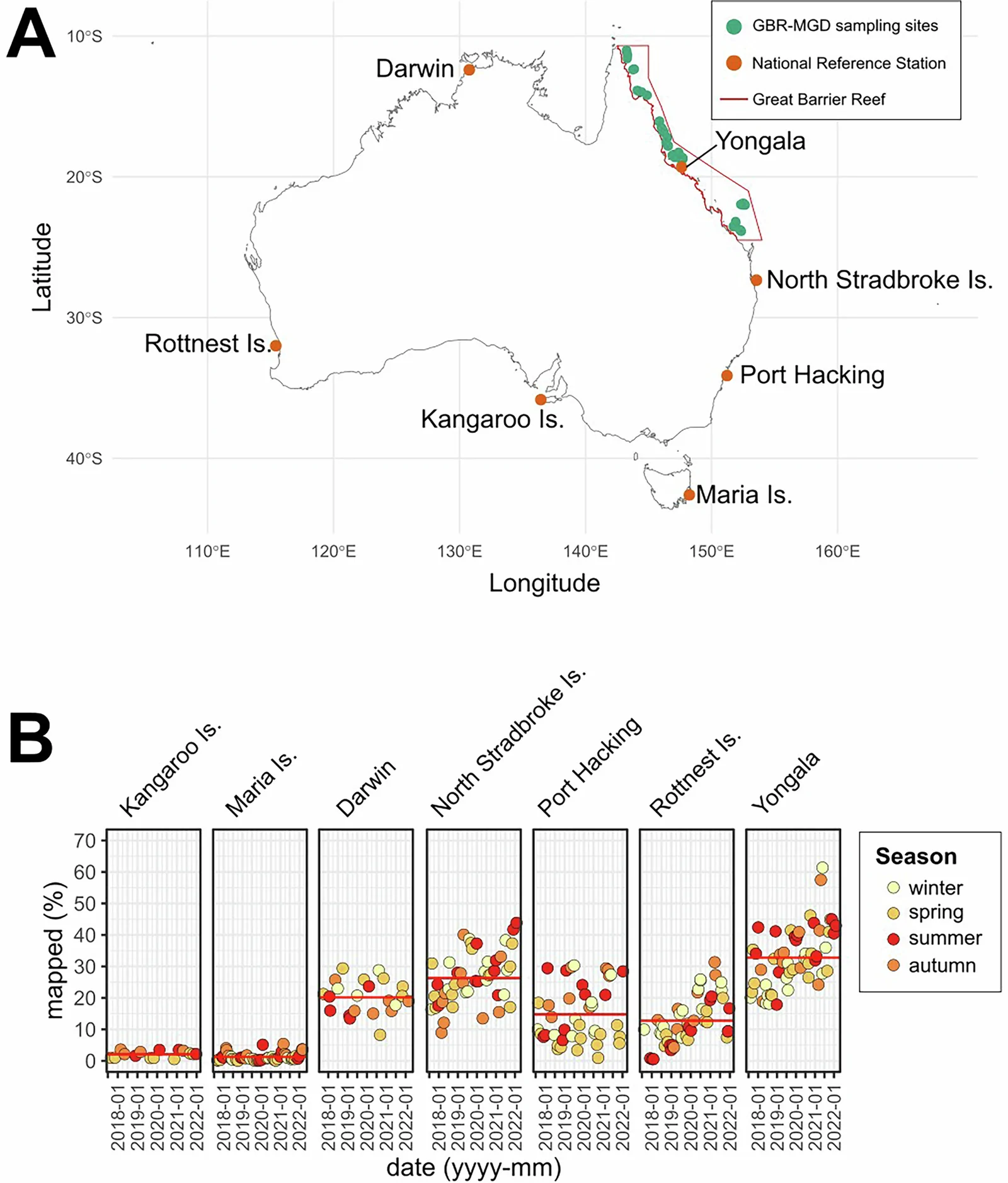
Read mapping rates of metagenomes from Australia’s seven National Reference Stations (NRS) to prokaryotic metagenome-assembled genomes (pMAGs) from the Great Barrier Reef Microbial Genomes Database (GBR-MGD). **A**, map of Australia showing location of GBR-MGD sampling sites and the seven NRS^91^. **B**, percent reads from NRS metagenomes mapped to GBR-MGD pMAGs (dereplicated at 95% average nucleotide identity). Each circle represents one metagenome data set coloured to indicate season. Red lines represent average % mapped reported on the top of the respective panels. The 272 NRS metagenomes were provided by Andrew Bissett (CSIRO Australia) and are available through the Bioplatforms Australia data portal (https://data.bioplatforms.com/).

Species-level dereplication (≥95% average nucleotide identity (ANI)) of the final set of GBR-MGD pMAGs resulted in a set of 876 unique species (Supplementary Table 6). To determine the novelty of the GBR-MGD data, we compared all pMAGs to the approximately 32,000 pMAGs (≥50 quality based on CheckM1 or CheckM2) present in the Ocean Microbiomics Database^4^ (OMD), revealing that 584 of the GBR-MGD species (66.7%) were not previously represented (<95% ANI to OMD sequences) (Supplementary Table 8). These may therefore represent either newly recognized marine species potentially specific to the GBR or taxa that are present elsewhere but are not represented in public databases owing to difficulties with assembly. Notably, of the 328 species clusters with members from both the GBR-MGD and OMD, GBR-MGD pMAGs were the best representative 65% of the time (213 clusters) based on CoverM Cluster scoring criteria, which take into account genome completeness, contamination and number of contigs in each pMAG. Thus, the GBR-MGD comprises a wealth of new species diversity captured in high-quality pMAGs. Mapping of the GBR-MGD Illumina reads from all samples to the dereplicated pMAGs (Supplementary Table 6) showed that an average of 47 ± 11% (maximum 66%) of the community was represented by prokaryotic taxa. This result exceeds previous read-mapping statistics of marine metagenomes against large prokaryote genome databases, including *Tara* Oceans (2,631 MAGs; 7% average mapping)^1^, GEM^26^ (8,578 MAGs; approximately 20% average mapping), single amplified genome (SAG)-only Global Ocean Reference Genomes Tropics Database^27^ (20,288 SAGs; approximately 20–40% average mapping), and is nearly on par with the OMD^4^, a gold standard aggregated set of >35,000 MAGs and SAGs from all previous marine studies (approximately 58% average mapping for the 0.2–3 µm size fraction). Mapping of the GBR-MGD Illumina reads to OMD MAGs and SAGs resulted in an average 40.4 ± 7.7% (maximum 53.7%) of the GBR community represented by OMD genomes, indicating that the 876 GBR-MGD pMAGs capture regional microbial diversity not yet present in the OMD. Additionally, average read mapping rates of seawater metagenomes from seven Australian National Reference Stations to the GBR-MGD pMAGs ranged from 1.3% to 32.8% (Extended Data Fig. 2), indicating that seawater microorganisms on the GBR are distinct compared with these marine monitoring sites across Australia.

**Fig. 3:**
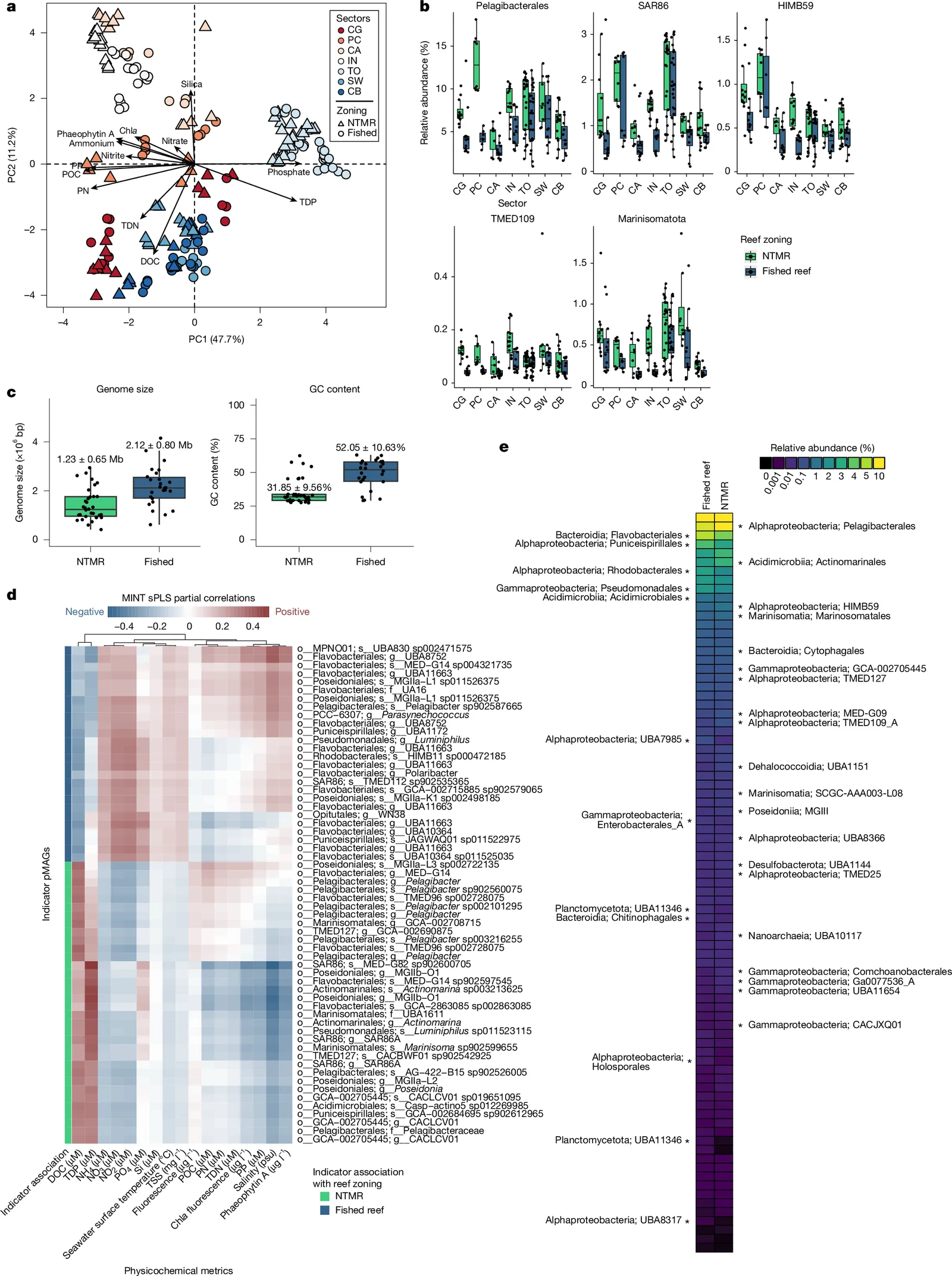
Microbial community composition on the GBR and indicators of reef protection zoning. **a**, Principal component analysis visualization of microbial community composition (*n* = 48 reef sites × 4 replicates). Chl*a*, chlorophyll *a*; DOC, dissolved organic carbon; PN, particulate nitrogen; POC, particulate organic carbon; PP, particulate phosphorus; TDN, total dissolved nitrogen; TDP, total dissolved phosphorus. **b**, Relative abundances of indicator taxa enriched in NTMRs (*n* = 23) compared with Fished reefs (*n* = 25) across the seven surveyed GBR sectors. **c**, Average genome size and GC content of the top 60 most influential indicator pMAGs (95% ANI dereplicated) from NTMR (*n* = 34 indicator pMAGs) and Fished (*n* = 26 indicators) reefs. Values quoted directly above the box plots are mean ± s.d. **d**, Heat map of MINT sPLS partial correlation coefficient values for each indicator pMAG showing positive (red) or negative (blue) correlation with seawater physicochemical variables. Si, silica; TSS, total suspended solids. **e**, Heat map showing microbial community composition of NTMRs (*n* = 23) versus Fished reefs (*n* = 25) based on relative abundances at order ranks. Asterisks indicate statistical association (*q* < 0.05, modelled using generalized linear models implemented in MaAsLin 3; *P* values generated using two-tailed tests and corrected for multiple comparisons with Benjamini–Hochberg false discovery rate) with either NTMRs (right side of heat map) or Fished reefs (left side of heat map). In box plots (**b**,**c**), centre lines represent median values, top and bottom box boundaries represent top and bottom quartiles, respectively, whiskers extend to 1.5× interquartile range, and minima and maxima are represented by data points.

Microbial community composition of the GBR differed across the seven sectors surveyed (adjusted *P* < 0.05, pairwise permutational multivariate analysis of variance (PERMANOVA); Fig. 3a), although a single *Parasynechococcus* species (sp002724845) was dominant in six of the sectors (8.5% to 14.7% average relative abundance) (Supplementary Table 6) and adjacent sectors tended to be compositionally similar (Fig. 3a). Communities in the seventh sector (TO) were distinct and significantly (adjusted *P* < 0.1, MaAsLin 3) enriched in *Prochlorococcus*_A sp003278705 (4.5% average relative abundance versus 0.5% in other sectors), *Pelagibacter* sp902612605 (0.6% versus 0.2%) and *Actinomarina* sp003213625 (0.5% versus 0.3%) (Supplementary Table 9). To determine potential drivers of the observed compositional variation, we compared two of the most divergent sectors, TO and IN. Water chemistry data fitted to community composition indicated that TO communities (Fig. 3a) were associated with fivefold higher phosphate and sixfold lower ammonium concentrations than IN (Supplementary Table 10). This was reflected in their metagenomes, with IN having higher abundances of Pst phosphate transport system and carbon-phosphorus lyase complex genes than TO (adjusted *P* < 0.1, DESeq2; Supplementary Table 11). These proteins facilitate the import of inorganic phosphate^28^ and metabolism of phosphonate compounds as a source of phosphate^29^, respectively, under phosphate-limiting conditions. Conversely, nitrate reductases were less abundant in IN compared with TO (Supplementary Table 11), possibly linked to inhibition of these enzymes by ammonium^30^.

## Microbial indicators of reef zoning

**Extended Data Fig. 3:**
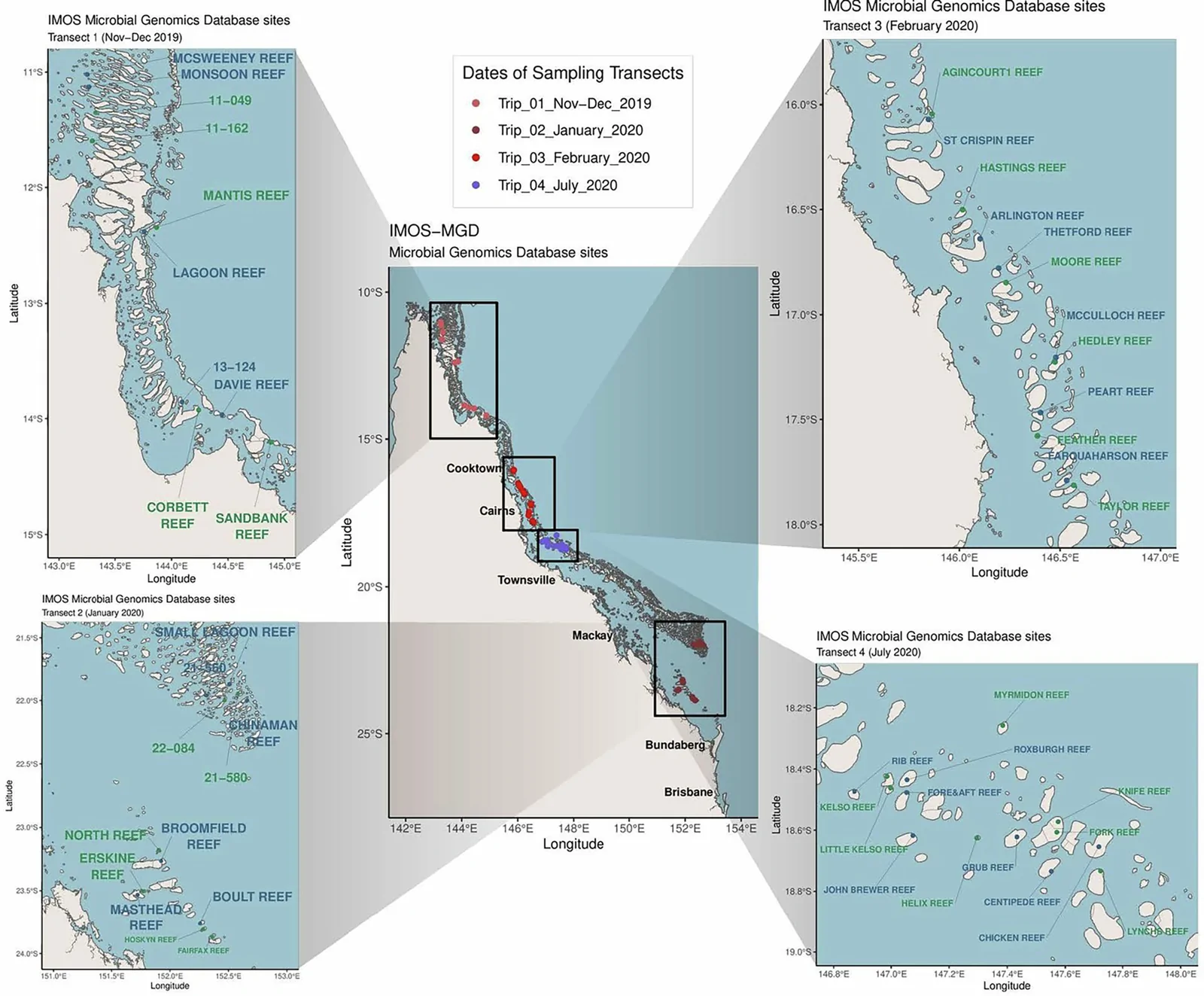
Site map showing Fished and No-Take Marine Reserve (NTMR) reefs designated under the 2004 Great Barrier Reef (GBR) Marine Park Authority (MPA) management plan. Reefs names in green font indicate NTMR reefs, blue font indicates Fished reefs. See GBR zoning maps for more information, available via the GBR MPA website https://www2.gbrmpa.gov.au/access/zoning/zoning-maps.

**Extended Data Fig. 4:**
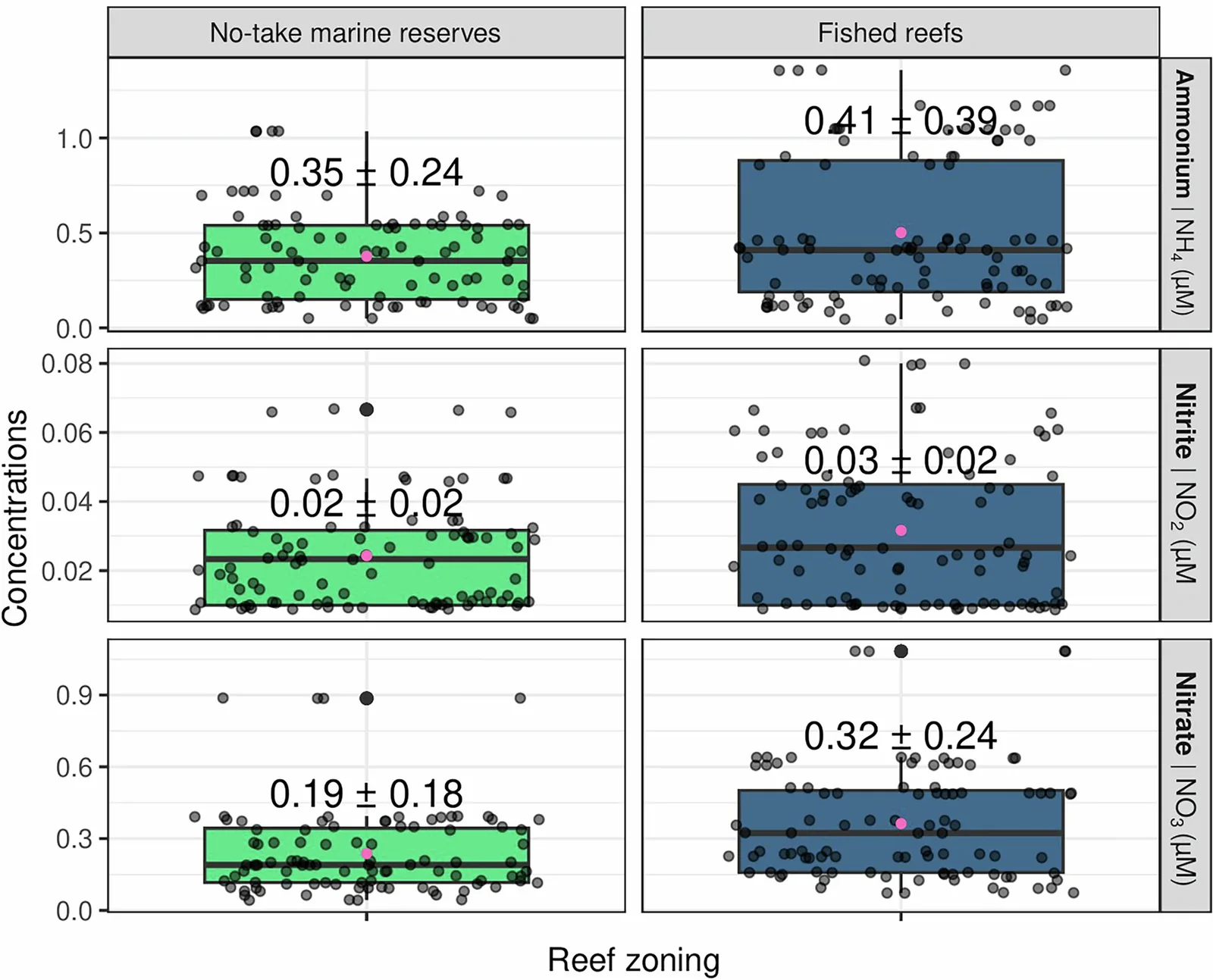
Dissolved nitrogen concentrations at No-Take Marine Reserve (NTMR) and Fished reefs. Three measurements with taken at each reef using three of the four water samples collected (n = 69 for NTMRs, n = 75 for Fished reefs). NTMR reefs (n = 23) are represented in green, Fished reefs (n = 25) in blue. Red dots represent average values, while the thick middle line, upper and lower box boundaries represent median, upper and lower quartiles, respectively. Whiskers extend to 1.5x interquartile range. Values quoted above the individual box plots are mean ± standard deviation.

Marine protected areas are refuge zones that were created to protect sensitive reef-dwelling animals from human disturbance. When strictly enforced, such areas are known to be effective tools for reef conservation that can buffer the negative effects of fishing and potentially marine heatwaves on sensitive species^31^. Consequently, the GBR Marine Park Authority has categorized reefs into zones^32^ that are open to fishing (Fished), and No-Take Marine Reserves (NTMRs), which are closed to fishing to protect ecosystems and maintain stable populations of commercially harvested fish (Supplementary Table 10). Marine microbial communities have been shown to shift rapidly and predictably in response to environmental change such as nutrient loads and temperature, which could be leveraged to inform management decisions^33^. To test whether the GBR-MGD can predict reef protection status, we analysed the abundances of prokaryotic, viral and eukaryotic MAGs and contigs (Methods, ‘Final GBR-MGD database compilation’) from co-located Fished and NTMR reefs (Extended Data Fig. 3) using multivariate integration sparse partial least squares discriminant analysis (MINT sPLS-DA) to perform feature selection controlling for sector, followed by classification with random forest. This approach predicted protection status with 74.6 ± 1.4% accuracy (mean ± s.d.), suggesting an ecosystem-wide effect that extends into the surrounding seawater to influence microorganisms. As prokaryotic communities are more easily profiled (for example, for development of environmental assessment tools^34^) and have been better characterized compared with viruses and eukaryotes, we repeated this analysis with pMAGs alone, resulting in 71.4 ± 0.9% accuracy (mean ± s.d.). Notably, the majority of pMAGs indicative of NTMR status belong to Pelagibacterales (7.9% versus 5.4% average relative abundance in NTMR versus Fished zones), SAR86 (1.6% versus 1.1%), HIMB59 (0.6% versus 0.5%), TMED109 (0.1 % versus 0.06%) and Marinisomatota (0.6% versus 0.4%) (Fig. 3b), all identified in the present study as lineages displaying high strain heterogeneity and low GC content that are underrepresented in Illumina short-read sequence data (Fig. 2). Many of these taxa have undergone genome streamlining (reduced genome size and lower GC content; Fig. 3c) as an adaptation to oligotrophic habitats to reduce their requirement for nutrients^35–37^ (Fig. 3d). By contrast, the majority of taxa indicative of Fished reefs have GC contents above 45% with no evidence of genome streamlining, including members of the genera UBA11663 and UBA8752, species UBA10364 sp003445735 in the class Bacteroidia, the family Halieaceae in the Gammaproteobacteria, and the species HIMB11 sp000472185 in the Alphaproteobacteria. In fact, at 60%, 63% and 45% average GC respectively, the UBA11663, UBA8752 and UBA10364 pMAGs have GC contents 9 to 27% higher than the average member of the Bacteroidia (36%) from the GBR-MGD (Supplementary Table 7). The association of NTMR reefs with streamlined, low-GC taxa that require less nitrogen for DNA synthesis^36–39^ than their higher-GC counterparts on Fished reefs suggests that protection status may influence reef nutrient levels. Leveraging nutrient measurement data taken alongside these microbial samples, we find that dissolved nitrogen (ammonia, nitrate and nitrite) is indeed lower in NTMR reefs compared with Fished reefs (Extended Data Fig. 4), suggesting that differences in nutrient levels between NTMR and Fished reefs select for taxa with different ecological and evolutionary strategies. Decreased fish densities and thus grazing are postulated to result in increased macroalgae, which in turn release nutrients that may account for higher nutrient levels in Fished reefs^33,40–42^.

Although NTMRs were generally associated with prokaryotic oligotrophs and Fished reefs with copiotrophs (Fig. 3e and Supplementary Table 12; false discovery rate-corrected *P* < 0.05, MaAsLin 3), the enrichment of specific taxa was region-dependent. For example, several oligotrophic *Pelagibacter* species were only enriched in IN NTMRs whereas *Prochlorococcus*_A was the most abundant genus in TO NTMRs. Conversely, inferred copiotrophic species such as *Luminiphilus* sp011524755 (order Pseudomonadales) were enriched in IN Fished reefs and MED-G52 sp001627375 (order Rhodobacterales) enriched in TO Fished reefs^43^. This regional specificity was also reflected by limited overlap of the most abundant species (3 out of 20) that distinguish reef protection zoning in each sector (adjusted *P* < 0.1, MaAsLin 3) (Supplementary Table 13). Regional specificity was also reflected to some extent by enrichment of different gene families in different sectors (Supplementary Tables 14 and 15). For example, adenosine triphosphate-binding cassette transport systems for inositol-phosphate (K17238, K17239 and K17240) were enriched in IN NTMRs, and transport systems for polar amino acids, spermidine and putrescine (K02029, K02052 and K02053) were enriched in TO NTMRs (adjusted *P* < 0.1, DESeq2), broadly indicative of low nutrient availability but probably indicating regional differences in available nutrients. Similarly, different genes suggestive of copiotrophic conditions were enriched in the different sectors. For example, in IN Fished reefs genes linked to the degradation of aromatic compounds such as phenylacetyl-CoA epoxidase (*paaABCDE*, K02609–K02613; phenylacetate degradation pathway) and *pcaDFG* (K00448, K01055 and K07823; β-ketoadipate pathway) were enriched, and in TO Fished reefs phenol 2-monooxygenase (K03380) and 4-methoxybenzoate monooxygenase (K22553) were enriched. In addition, TO NTMRs were enriched in several *Kyanoviridae* protein orthologues (Supplementary Table 15), probably linked to the high relative abundance of *Prochlorococcus*^44^ (~4.5% relative abundance) in this sector. Together, these data indicate that whereas NTMR zoning results in broad enrichment of oligotrophic taxa, region-specific differences are more nuanced and highlight that markers tailored to regions could increase predictive power for reef management. More samples from NTMR and Fished reefs in each sector will be needed to test this idea.

## Recovery of DNA viruses

**Extended Data Fig. 5:**
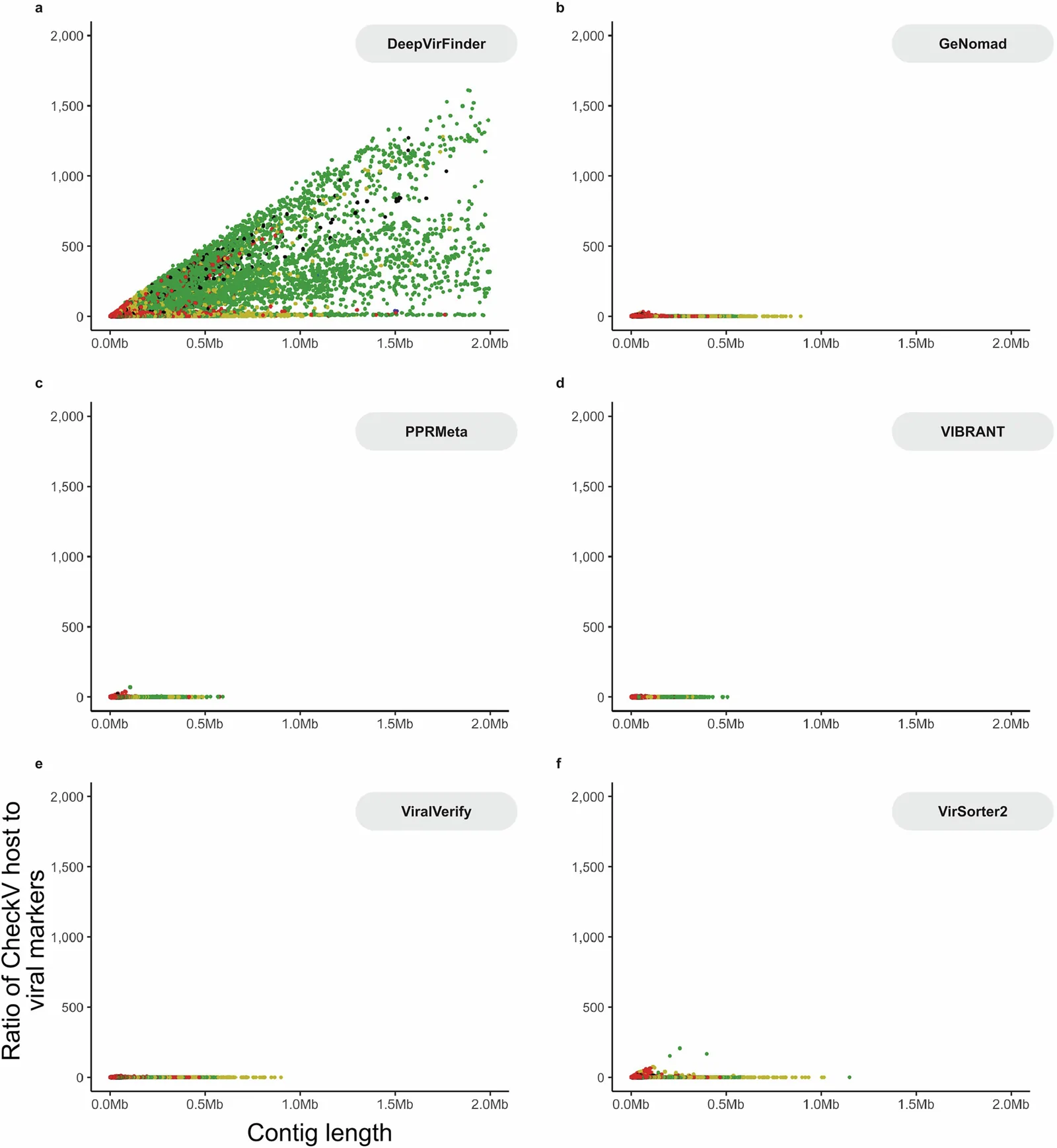
Ratio of CheckV host to viral markers vs contig length in hybrid assemblies. Dotplots show the ratio of CheckV host to viral marker genes in contigs predicted to be viral by a) DeepVirFinder, b) GeNomad, c) PPRMeta, d) VIBRANT, e) ViralVerify, and f) VirSorter2 in hybrid Nanopore-Illumina assemblies from the GBR-MGD. Colours indicate the assigned CheckV completeness label of Complete (blue), High-Quality (green), Medium-quality (yellow), Low-Quality (red), and Not Determined (black).

**Extended Data Fig. 6:**
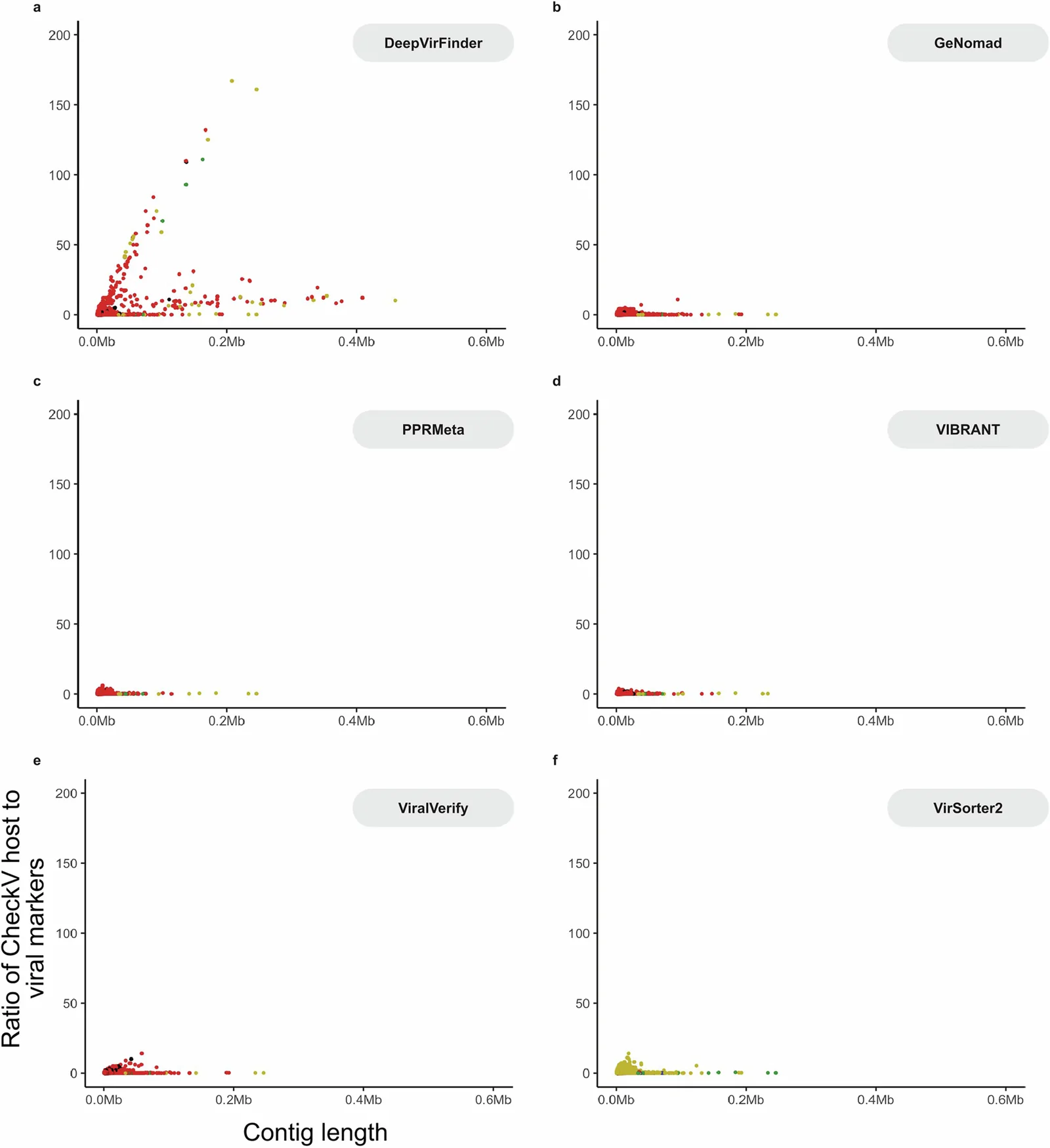
Ratio of CheckV host to viral markers vs contig length in Illumina-only assemblies. Dotplots show the ratio of CheckV host to viral marker genes in contigs predicted to be viral by a) DeepVirFinder, b) GeNomad, c) PPRMeta, d) VIBRANT, e) ViralVerify, and f) VirSorter2 in Illumina-only assemblies from the GBR-MGD. Colours indicate the assigned CheckV completeness label of Complete (blue), High-Quality (green), Medium-quality (yellow), Low-Quality (red), and Not Determined (black).

**Extended Data Fig. 7:**
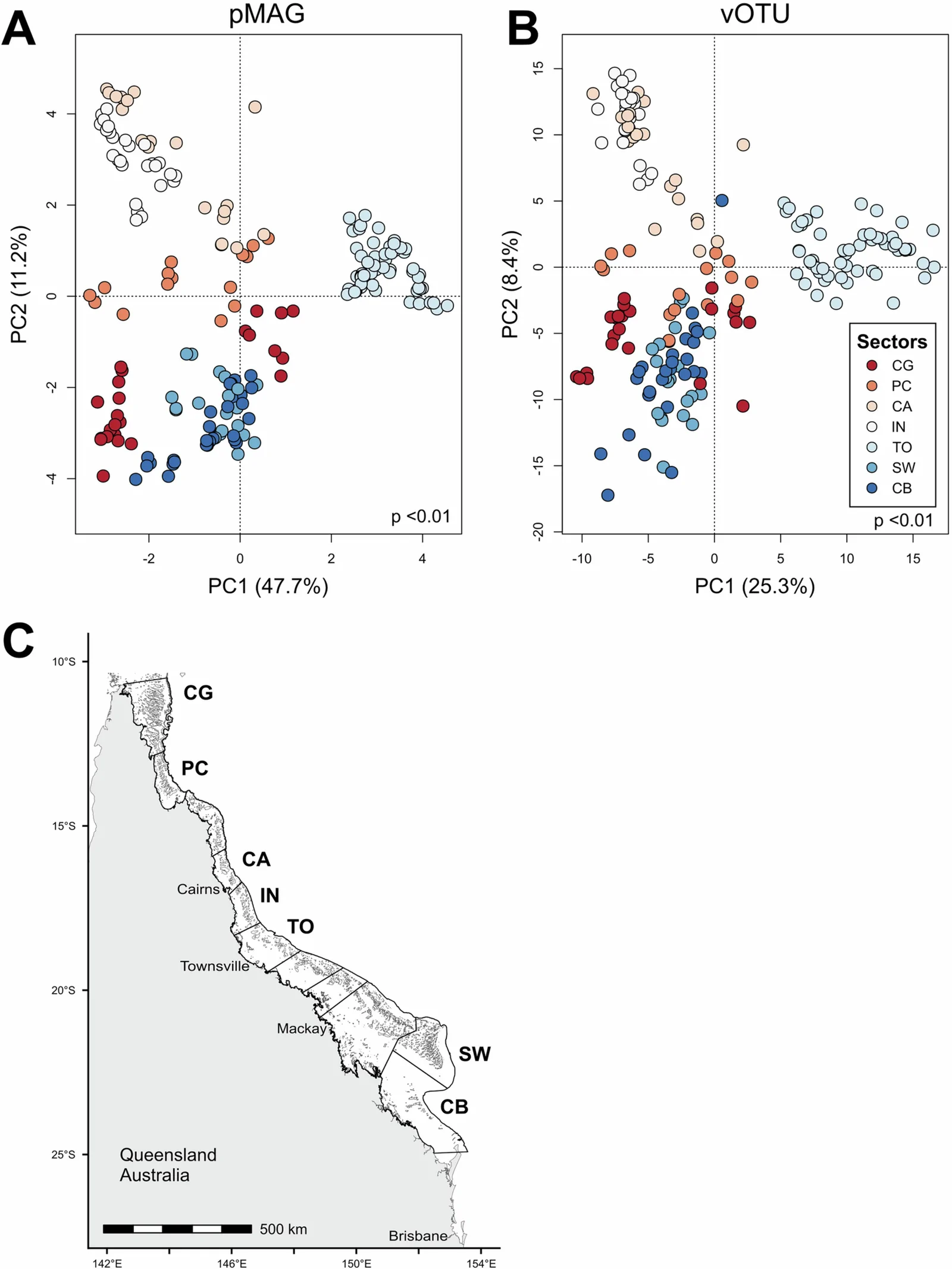
Community composition of prokaryotic metagenome-assembled genomes (pMAGs) and virus operational taxonomic units (vOTUs) on the Great Barrier Reef (GBR). **A**, Principal component analysis of community composition of pMAGs. **B**, Principal component analysis of community composition of vOTUs. **C**, location of GBR sectors sampled in this study. Circles in panels A and B are coloured by GBR sector; refer to panel C for sector locations. Community composition significantly differed by sector (p < 0.01, PERMANOVA). CG: Cape Grenville, PC: Princess Charlotte Bay, CA: Cairns, IN: Innisfail, TO: Townsville, SW: Swain, CB: Capricorn Bunker.

Viruses are the most abundant biological entities in marine environments, and have a large impact on marine ecology through interactions with their prokaryote and eukaryote hosts. For example, it is estimated that viruses kill 20% of marine microbial biomass per day, representing a substantial carbon turnover^45^. Current state-of-the-art viral detection tools have almost exclusively been benchmarked on short-read assemblies, and are known to be sensitive to contig length^46,47^. To assess their use on long-read assemblies, we first tested six widely used metagenomic viral prediction tools to identify viruses in the GBR-MGD—GeNomad, Virsorter2, ViralVerify, VIBRANT, PPR-Meta and DeepVirFinder—using CheckV to assess the number of complete viruses predicted by each tool. We further plotted the ratio of prokaryotic host to viral marker genes identified by CheckV within each putative viral contig to detect spurious assignments. DeepVirFinder showed substantially more false positives than the other tools tested, with the proportion of bacterial marker genes among putative viral contigs increasing with contig length, indicating bacterial origin (Extended Data Figs. 5 and 6). When these bacterial contigs were assessed by CheckV, longer contigs were increasingly likely to be designated as complete viral genomes, despite having a high ratio of bacterial to viral markers (Extended Data Figs. 5 and 6). We note that CheckV is not designed to identify viral versus non-viral sequences, but highlight this result because it is common practice to filter assemblies based on CheckV quality scores that could result in extensive misidentification of viral auxiliary metabolic genes if DeepVirFinder is applied to long-read assemblies. We therefore excluded DeepVirFinder from our pipeline as unsuitable for use with long-read metagenomes, and the remaining five tools were used in concert to produce a consolidated dataset of 808,585 single contig viral MAGs (vMAGs) from across the 48 GBR-MGD reefs, representing 362,802 species-level (95% ANI) viral operational taxonomic units (vOTUs) (Supplementary Table 16). Read mapping against the vOTU representatives accounted for 4–27% of each metagenome (averaging 13 ± 4%), highlighting that viruses comprise a substantial proportion of GBR seawater metagenomes^45,48^. The overall vOTU community composition significantly differed across sectors (*P* < 0.01, PERMANOVA) and closely tracked the prokaryotic communities (*P* < 0.01, Procrustes rotation; Extended Data Fig. 7).

The majority of vOTUs were classified as *Caudoviricetes* (67% of sequences), of which only 6% were resolved below this class. This included the families *Kyanoviridae* (2.8%) encompassing the T4-like cyanophages that commonly infect *Prochlorococcus*^44^ and *Synechococcus*^49^; *Autographiviridae* (1.4%), common phages of *Prochlorococcus*, *Synechococcus* and *Pelagibacter*^50–53^; and the order *Crassvirales* (1%; see below). A further 6% of the vOTUs were classified as giant viruses belonging to the *Nucleocytoviricota*, which probably reflects our ability to recover low-GC genomes characteristic of this phylum. The remaining vOTUs (27%) were unclassified, which may indicate bona fide marine viral novelty or difficulties in discriminating viruses from other genetic elements. Therefore, we provide the CheckV and GeNomad marker gene frequency tables to enable users to filter vMAGs based on their own criteria (Supplementary Tables 17–19).

## Recovery of marine *Crassvirales*

**Fig. 4:**
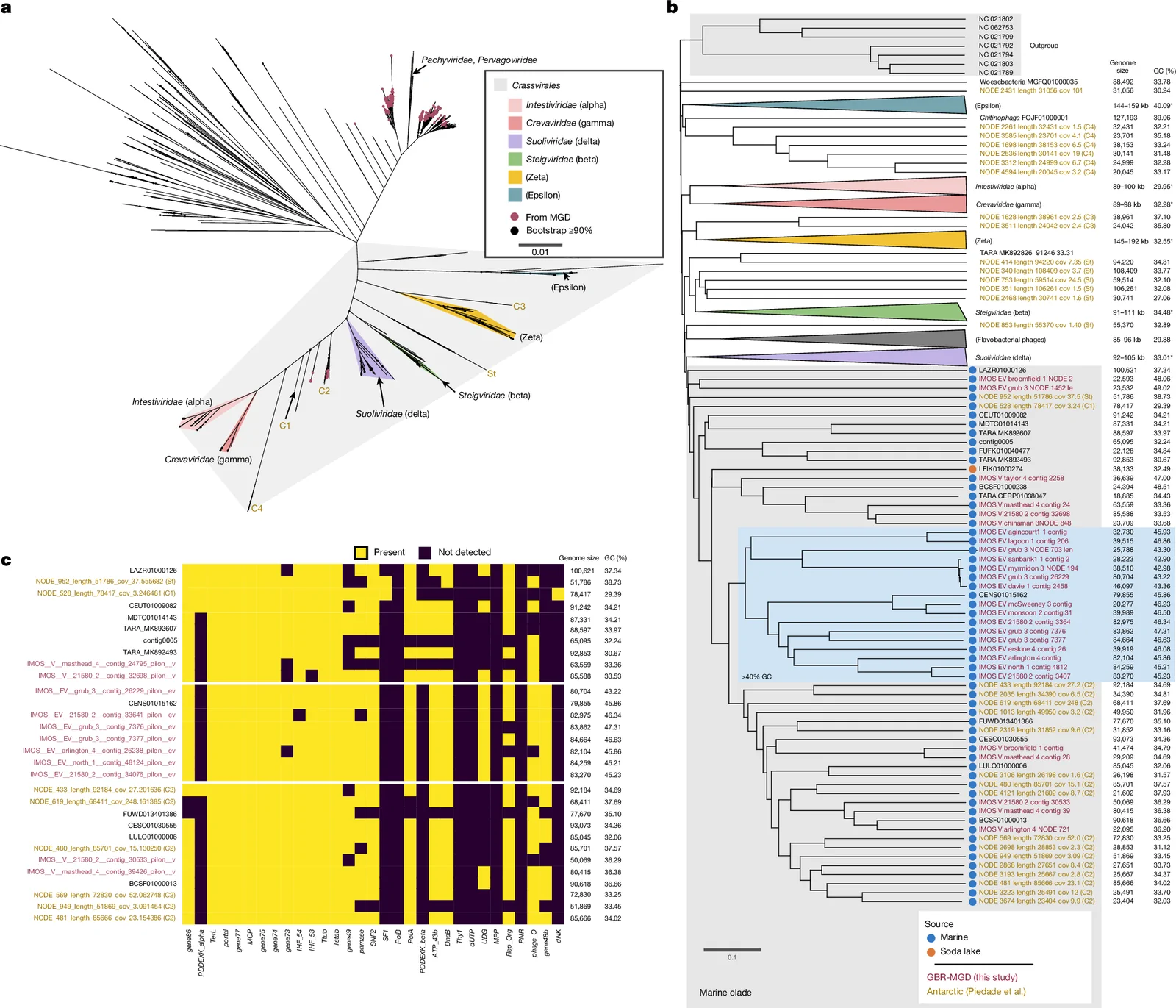
Expansion of the marine *Crassvirales.* **a**, TerL tree of *Crassvirales* and other *Caudoviricetes* showing placement of GBR-MGD vMAGs represented by red circles (MGD) in relation to existing *Crassvirales*. Families are labelled with International Committee on Taxonomy of Viruses (ICTV) names along with the previously proposed alpha, beta, gamma, delta, epsilon and zeta group names in parentheses^59^. C1–C4 and St groups are as proposed in Piedade et al.^62^. **b**, Whole-genome phylogeny (ViPTree) of GBR-MGD *Crassvirales* vMAGs larger than 20 kb (*n* = 27). **c**, Pattern of *Crassvirales* gene conservation in vMAGs and genomes within the marine clade. Only genomes larger than 50 kb are shown. Genes included here were previously identified by Yutin et al.^59^. ATP_43b, AAA domain ATPase; DnaB, phage replicative helicase DnaB family; dNK, deoxynucleotide monophosphate kinase; dUTP, dUTPase; UDG, uracil-DNA glycosylase; gene48b, phage endonuclease I; gene49, uncharacterized protein gene 49; gene73, putative structural protein gene 73; gene74, putative structural protein gene 74; gene75, putative structural protein gene 75; gene77, putative structural protein gene 77; gene86, putative structural protein gene 86; IHF_53, IHF subunit gene 53; IHF_54, IHF subunit gene 54; MCP, major capsid protein; MPP, metallophosphatase; PDDEXK, PD-(D/E)XK family nuclease; phage_O, bacteriophage replication protein O; PolA, DNA polymerase family A; PolB, DNA polymerase family B; portal, portal protein; primase, DnaG family primase; Rep_Org, replisome organizer protein; RNR, ribonucleotide reductase; SF1, SF1 helicase; SNF2, SNF2 helicase; TerL, terminase large subunit; Thy1, thymidylate synthase; Tstab, tail stabilization protein (P22 gp10-like); Ttub, tail tubular protein (P22 gp4-like). *Crassvirales* genomes recovered in this study are represented by red circles in **a** or red labels in **b**,**c**; genomes and groups from Piedade et al.^62^ are represented in yellow (C1–C4 and St in **a**, genome labels in **b**,**c**). Colour labels of the *Crassvirales* clades are consistent across **a**,**c**.

Crassphages were first identified in cross assemblies of human gut metagenomes^54^ and are currently recognized as the most abundant phage in the human gut^55,56^. Although members of the *Crassvirales* have since been identified in a range of additional environments such as primate and termite gut samples, terrestrial systems, groundwater and marine habitats^57–60^, *Crassvirales* from these habitats remain poorly characterized. Within the GBR-MGD, 312 vMAGs were classified by GeNomad as *Crassvirales*, of which 167 contained a terminase large subunit marker gene (*terL*) containing at least 400 amino acid residues. However, phylogenetic placement in a TerL tree containing representative genomes from the class *Caudoviricetes* indicated that most of these sequences (155, 93%) are members of sister clades to the *Crassvirales*, the families *Pachyviridae* and *Pervagoviridae* (Fig. 4a), which primarily comprise free-living marine flavobacterial phages^61^. To confirm the placement of the GBR-MGD *Crassvirales* within the order, we constructed whole genome phylogenies including all putative *Crassvirales* GBR-MGD vMAGs more than 20 kb in length and select reference genomes from the *Caudoviricetes*, including the *Pachyviridae* and *Pervagoviridae* (Fig. 4b). This tree also contained recently discovered *Crassvirales* sequences from Antarctic seawater^62^, providing an opportunity to assess the phylogenetic distribution of the *Crassvirales* across global oceans and between tropical coral reefs and Antarctic waters (Fig. 4b). Four distinct clades of Antarctic *Crassvirales*, C1–C4, have previously been proposed^62^. Notably, GBR-MGD *Crassvirales* are part of a larger monophyletic clade of exclusively marine sequences that includes the C1 and C2 clades (Fig. 4a). We confirm that all members of this marine lineage contain the full complement of core *Crassvirales* genes (Fig. 4c). Of note, most GBR-MGD *Crassvirales* form a separate clade within the marine lineage characterized by a higher GC content (average 45% versus 34%; Fig. 4b). However, several cluster with C2 genomes indicating that the C2 clade is not limited to the Antarctic but is also present in Australian tropical reefs, revealing a wider geographical distribution and temperature range (Fig. 4b). Similar to animal-associated *Crassvirales*, the marine lineage, both Antarctic and GBR, are predicted to infect Bacteroidota hosts^60^, suggesting that this association is common to this viral order and that marine *Crassvirales* could be linked to marine carbon fluxes through interactions with their hosts^41^. Further work is necessary to delineate the global distribution and ecological characteristics of marine *Crassvirales*.

## Recovery of picoeukaryote genomes

Marine phytoplankton are responsible for around half of all primary production on the planet^8^, and although picoeukaryotes typically make up a smaller fraction of the phytoplankton community compared with prokaryotes, they exhibit much higher growth and turnover rates and often account for the majority of primary productivity^63,64^. Despite their ubiquity in marine ecosystems^12,65^, much of our knowledge of these groups relies on analysis of a few cultured isolates, none of which are from coral reefs or Australian oceans^66–68^. Researchers have recently begun to recover genomes from uncultured representatives via metagenomics^12^. However, as picoeukaryotes have more complex genomes than their prokaryotic counterparts, including multiple chromosomes, large genome rearrangements and more extensive repeat regions than prokaryotes, the few available short-read-based eMAGs are mostly fragmented and incomplete^12^.

**Fig. 5:**
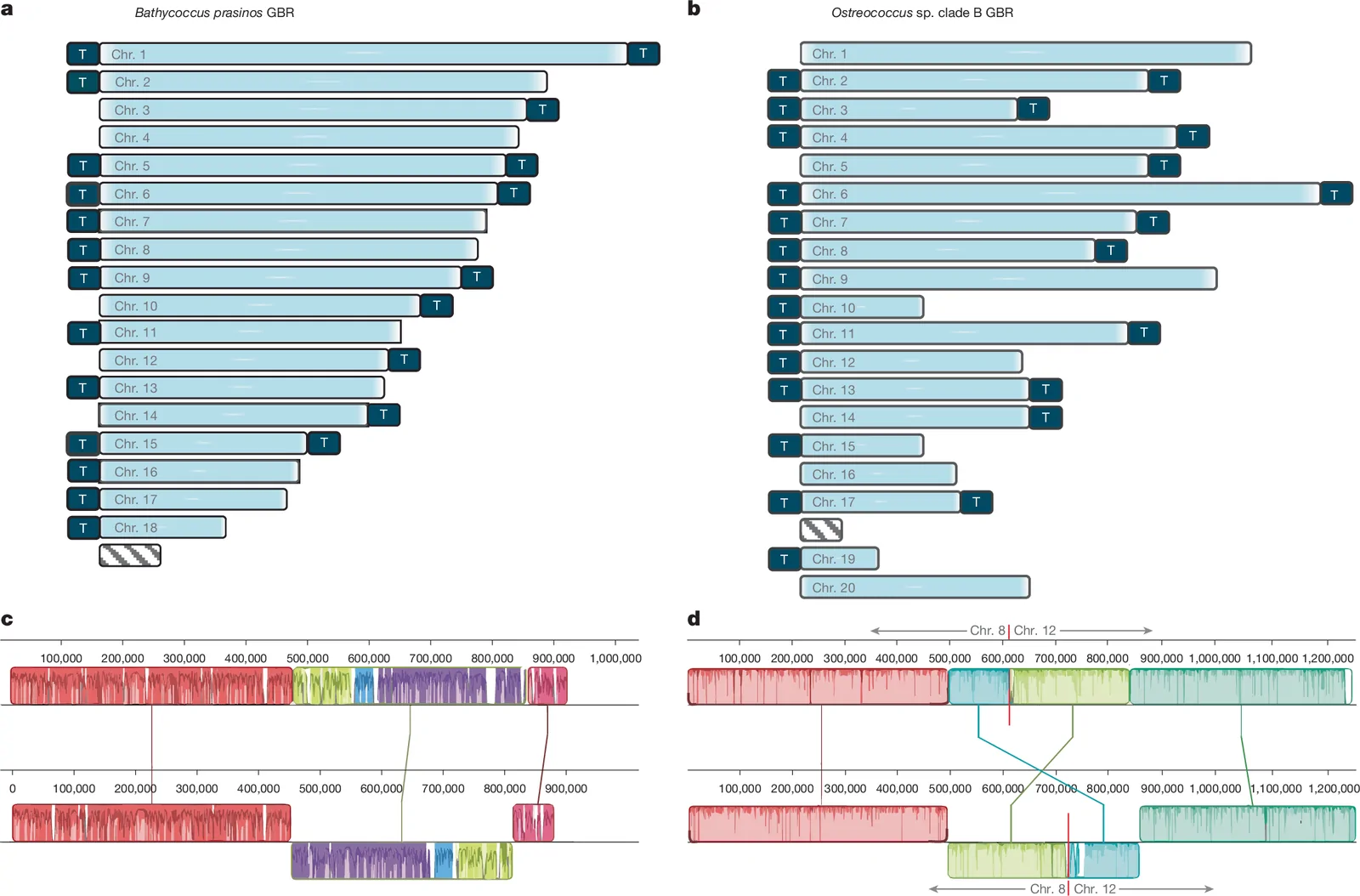
Chromosome-level eMAGs from marine picoeukaryotes. **a**, Composite illustration of the presence of telomeres (T) in GBR-MGD *Bathycoccus* eMAGs. Presence is noted in dark blue if telomeric repeats were found at the ends of chromosomes (Chr.) from at least one of the five eMAGs. **b**, Composite illustration of the presence of telomeres in GBR-MGD *Ostreococcus* sp clade B eMAGs. Presence is noted in dark blue if telomeric repeats were found at the ends of chromosomes from at least one of the 15 eMAGs. **c**, Sequence alignment of chromosome 4 for *Bathycoccus prasinos* RCC1105 (top) and GBR-MGD *Bathycoccus* variant 2 (bottom) from Grub Reef showing a large approximately 350 kb inversion in variant 2 compared with the reference. **d**, Sequence alignment of chromosomes 8 and 12 from *Ostreococcus* sp. clade B RCC809 (top) and GBR-MGD *Ostreococcus* sp clade B (bottom) from Masthead Reef showing an approximately 350 kb translocation between the ends of the two chromosomes. The striped chromosomal element in **a**,**b** represent the small outlier chromosome, which could not be recovered owing to its highly divergent sequence composition.

**Extended Data Fig. 8:**
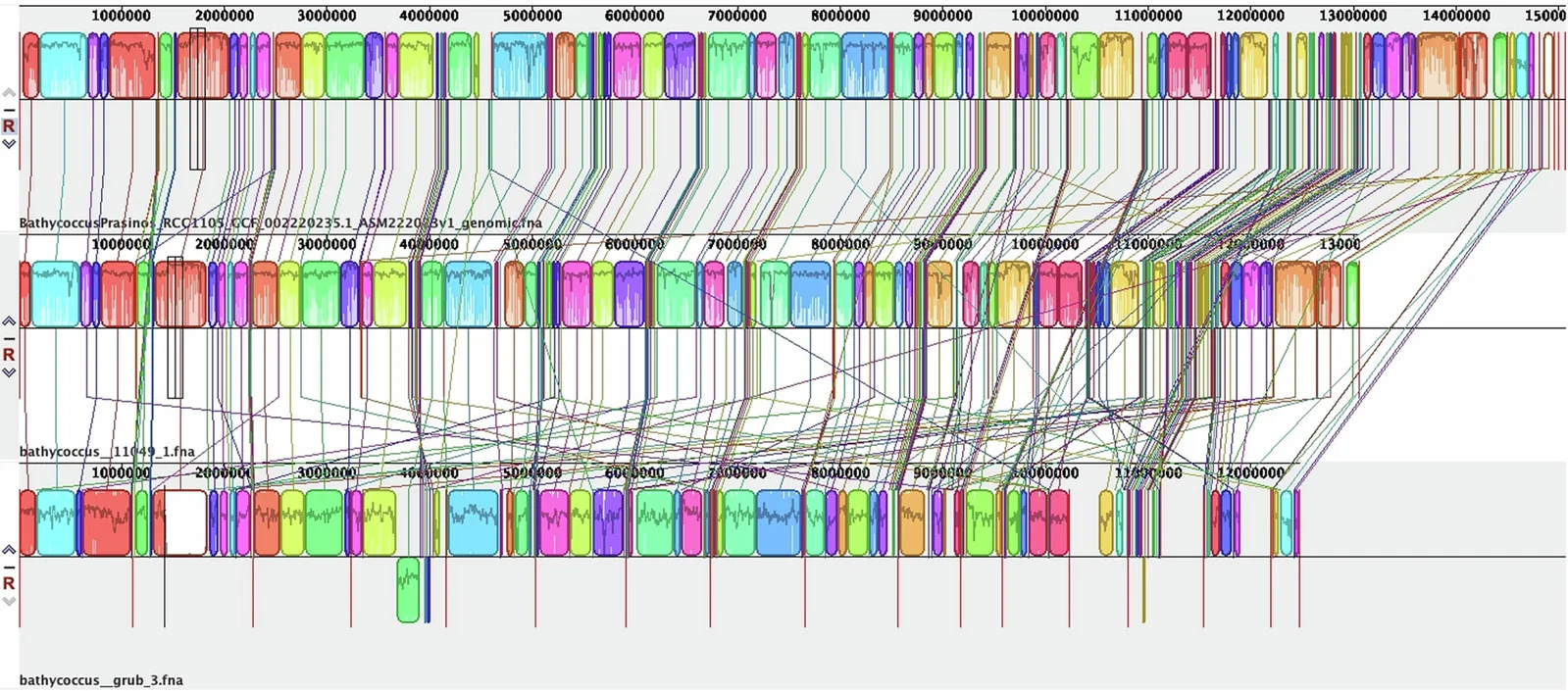
Whole genome alignment of GBR-MGD *Bathycoccus* vs *Bathycoccus prasinos* RCC1105. Alignment of the isolate reference genome from *Bathycoccus prasinos* RCC1105 (top row), *Bathycoccus prasinos* GBR_11049 from the GBR-MGD (middle), and *Bathycoccus prasinos* GBR_Grub from the GBR-MGD (bottom) using the Mauve aligner (progressiveMauve). Each chromosome is represented by one contig and vertical red lines represent contig boundaries. The small outlier chromosome (SOC) is not shown as it is highly divergent from the reference and could not be recovered.

**Extended Data Fig. 9:**
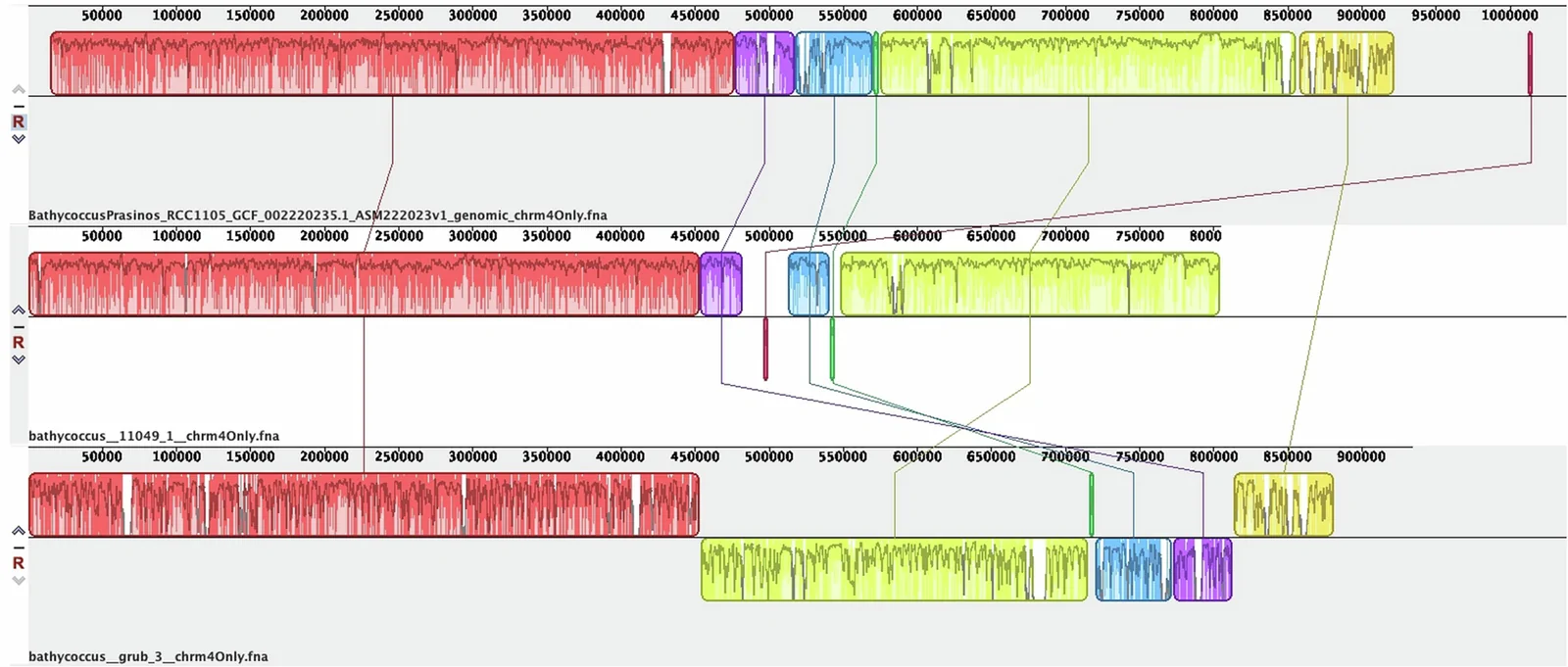
Alignment of *Bathycoccus prasinos* chromosome 4. Alignment of *Bathycoccus prasinos* RCC1105 (top row), *Bathycoccus prasinos* GBR_11049 from the GBR-MGD (middle), and *Bathycoccus prasinos* GBR_Grub from the GBR-MGD (bottom) using the Mauve aligner (progressiveMauve). Vertical red lines represent contig boundaries.

Using Nanopore long reads, we were able to obtain telomere-containing (Supplementary Table 20) chromosome-level eMAGs from the GBR for the picoeukaryote genera *Bathycoccus* and *Ostreococcus* (green algae in the order Mamiellales, phylum Chlorophyta), taxa that are ubiquitously distributed in the world’s oceans^63^ (Fig. 5a,b). In total, five *Bathycoccus* eMAGs were recovered and found to represent two distinct genome variants, BV1 and BV2, with BV1 being closely related to *Bathycoccus prasinos* RCC1105 (96% ANI) and BV2 representing a more distantly related lineage with only 82% ANI to RCC1105, along with a large approximately 350 kb inversion on chromosome 4 that is not present in BV1 (Fig. 5c and Extended Data Figs. 8 and 9). Mapping of the metagenomic reads to representatives of each variant (BV1, reef 11049; BV2, Grub Reef) revealed that they had similar relative abundance across most samples (average 0.07 ± 0.11% for BV1 and 0.08 ± 0.08% for BV2), except for samples collected from TO in which BV2 was about sevenfold more abundant (average 0.47%, maximum 2.4%; Supplementary Table 21). Previous marker gene-based studies suggest the existence of multiple *Bathycoccus* ecotypes that may represent different species^65,69^, though the genomic underpinnings of ecotype ecology are unclear and may be clarified through the recovery of additional chromosome-level genomes.

**Extended Data Fig. 10:**
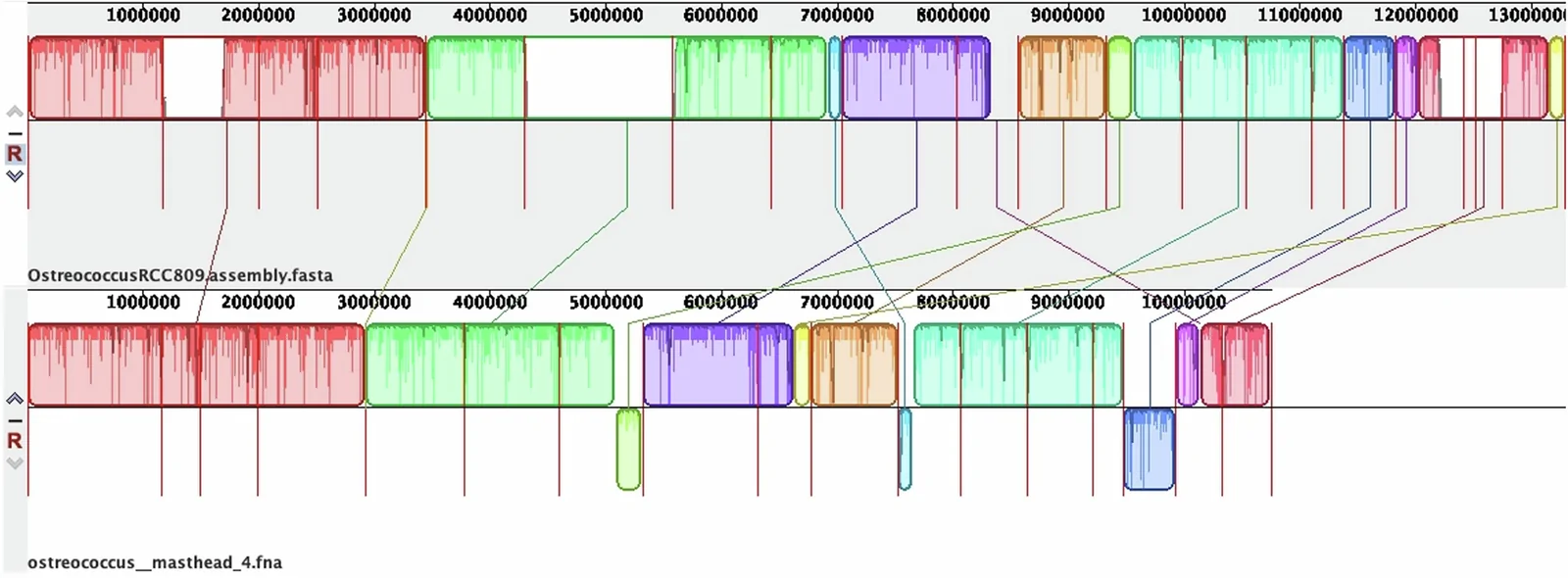
Whole genome alignment of GBR-MGD *Ostreococcus* clade B vs *Ostreococcus* clade B RCC809. Alignment of the isolate reference genome from *Ostreococcus* clade B RCC809 (top row) and *Ostreococcus* clade B from Masthead reef from the GBR-MGD (bottom) using the Mauve aligner (progressiveMauve). Each chromosome is represented by one contig and vertical red lines represent contig boundaries.

In contrast to *Bathycoccus*, all 15 GBR *Ostreococcus* eMAGs represent a single species that is closely related to *Ostreococcus* RCC809 (>99% ANI to *Ostreococcus* RCC809), a member of the clade B lineage, for which little genomic information is available. Despite high similarity to RCC809, all GBR *Ostreococcus* clade B eMAGs have an approximately 350 kb translocation between chromosomes 8 and 12 (Fig. 5d and Extended Data Fig. 10), indicating that high sequence similarity can hide major structural variation detectable via chromosome-level assemblies. After *Parasynechococcus* sp002724845, *Ostreococcus* clade B represents the second most abundant microbial species in the GBR-MGD (average 3.5%, maximum 30%; Supplementary Table 21). The ability to readily recover eMAGs with long reads, especially to chromosome-level quality, is a substantial advance that we hope will encourage others to explore uncultured eukaryotic dark matter.

## Discussion

Metagenomics has greatly enabled the study of microbial ecosystems by bypassing the cultivation bottleneck. However, marine ecosystems are unusual in that the genomes of many dominant microbial populations are conspicuously underrepresented in short-read MAG datasets. Prominent examples include the bacterial genera *Pelagibacter*, *Prochlorococcus* and *Synechococcus*, which are all underrepresented in international consortium-wide datasets. We show that this phenomenon is owing to limitations of short-read sequencing and assembly in handling the combination of high strain heterogeneity and low GC content of these taxa. By contrast, the Nanopore platform addresses both issues by being able to sequence low-GC templates and to traverse strain variable regions. However, Nanopore sequencing presents its own challenges, such as higher error rates in the raw reads and lower data output compared with Illumina platforms. Therefore, hybrid sequencing approaches that include long reads will produce the most comprehensive and high-quality genome inventory of marine microbial taxa using currently available technologies.

The GBR-MGD will be a highly valuable resource for marine researchers, not only because of its inclusion of dominant under-represented prokaryotic taxa, but also because it combines metagenomic data including high-quality prokaryote, viral and eukaryote genomes with seawater physicochemical measurements and metadata from the Australian Institute of Marine Science (AIMS) Long Term Monitoring Program (LTMP). We demonstrate the utility of this connection to explain the underlying physicochemical drivers of microbial community composition on the GBR and bioindicators of reef protection status. We expand the known diversity of marine *Crassvirales* and note that many of the GBR-MGD sequences form a high GC cluster, which is atypical for this viral order, and link *Crassvirales* to Bacteroidota that have important roles in nutrient cycling^70^ and, more recently, are gaining attention as emerging pathogens in marine ecosystems^71^. We also demonstrate that chromosome-level genome recovery is possible for microbial eukaryotes, which comprise up to 30% of pelagic GBR microbial communities.

We suggest that long-read sequencing should become a standard part of metagenomic analyses of marine microbial communities, as many key populations are poorly accessible via short-read sequencing alone. As low-GC taxa and strain heterogeneity are not limited to marine environments^24,72–74^, we predict that the sequencing limitations observed in this study will affect metagenomic analyses of other ecosystems that could be addressed through the incorporation of long reads. Finally, we suggest that integration of long-read metagenomics (biology) and physicochemistry measurements (nutrients) with reef monitoring will provide the necessary framework to enable adaptive reef management^34,75^.

## Methods

### Sample collection

Samples were collected under the permit G12/35236–1 issued by the Great Barrier Reef Marine Park Authority. Seawater samples were collected from 48 coral reefs spanning the length of the GBR, aligning with in situ coral surveys by the AIMS LTMP in 2019 and 2020 (Fig. 1). Four 5-l biological replicates of seawater were collected above the reef (2–10 m depth) at each site (total *n* = 192) in either Nalgene or Niskin bottles and placed on ice until return to the vessel. Each 5 l volume was independently filtered successively through 5-µm, 33 mm diameter cellulose acetate and 0.22-µm Sterivex-GP filters (Millipore). The Sterivex filters were then snap frozen and stored at −75 °C until DNA extraction.

In addition to metagenomics data, a total of 17 physicochemical variables were measured as part of this survey on 3 of the 4 replicates (5 l each) from each site, including ammonia (NH_4_^+^), nitrite (NO_2_^−^), nitrate (NO_3_^−^), total dissolved nitrogen, phosphate (PO_4_^3−^), total dissolved phosphorus, dissolved organic carbon, silicate, total suspended solids, chlorophyll *a* pigment concentration, phaeophytin A, particulate organic carbon, particulate nitrogen, particulate phosphorus, temperature, salinity and chlorophyll *a* fluorescence, using methods described in Terzin et al.^76^.

### DNA extraction

DNA extractions were carried out inside each 0.22-µm Sterivex filter by adding 1.8 ml filter-sterilized lysis buffer containing 50 mM Tris-HCL (pH 8.0), 40 mM EDTA (pH 8.0), 256 mg ml^−1^ sucrose and 18 µl lysozyme (100 mg ml^−1^). The filters were incubated with mild agitation for 1 h at 37 °C, followed by the addition of 20 µl proteinase K (20 mg ml^−1^) and further incubation and agitation for 1 h at 55 °C. The lysate was then ejected through the Sterivex into a 5 ml microtube and an equal volume of phenol:chloroform:isoamyl alcohol (IAA; 25:24:1) was added, mixed by inversion and centrifuged at 16,000*g* for 10 min. The aqueous phase was then recovered and an equal volume of chloroform:IAA (24:1) was added and mixed by inversion, then centrifuged for 10 min at 16,000*g*. DNA was precipitated by adding 1 ml isopropanol and recovered by centrifugation at 20,000*g* for 25 min and the supernatant discarded. The DNA was washed with 500 µl 70% ethanol, centrifuged for 10 min, the ethanol discarded and the pellet air-dried. Finally, 20 µl PCR water was added, incubated at 4 °C overnight to resuspend the DNA pellet and frozen at −20 °C.

### Illumina sequencing

All 191 replicates from the 48 sites were subjected to Illumina sequencing at the Centre for Microbiome Research (Brisbane, Australia). Libraries were prepared using the Nextera DNA Flex Library Preparation Kit (Illumina 20018705) according to manufacturer’s protocol. Libraries were pooled at equimolar amounts of 2 nM per library. The library pool was quantified in triplicates using a Qubit dsDNA HS Assay Kit (Invitrogen) and sequenced using an S4 2× 150 bp flow cell on a NovaSeq 6000. Additional ‘deep’ sequencing was carried out for one replicate from 27 of the 48 sites to a target depth of 40 Gbp for Nanopore polishing and hybrid assembly (Supplementary Tables 1 and 2).

### Nanopore sequencing, basecalling and quality control

The replicate chosen for Illumina deep sequencing was also submitted for long-read sequencing on Oxford Nanopore Technology’s (ONT) PromethION platform at the Australian Centre for Ecogenomics (Brisbane, Australia) sequencing facility. For ONT sequencing, DNA was first size-selected using the Circulomics SRE XS kit (PacBio, SKU 102-208-200) as this was shown in a small trial to increase total data output and read N50. Native barcoded libraries were prepared from the size-selected DNA following a genomic DNA by ligation protocol (Oxford Nanopore, LSK-109 with EXP-NBD104). Barcoded libraries were pooled with two samples per PromethION flow cell at equal concentration and the library pool was sequenced on a PromethION 24 (Oxford Nanopore) for a total of 72 h with a R9.4 flow cell using MinKNOW (v20.06.18) with default settings. We then performed rebasecalling of the fast5 files using the superaccuracy model with Guppy (v5.0.16) to generate higher accuracy raw reads before assembly. Finally, Porechop (https://github.com/rrwick/Porechop; v0.2.4) was used for barcode trimming using default settings.

### Hybrid metagenome assembly

Deep Illumina and nanopore reads for each of the 27 sites were assembled using the Aviary assembly pipeline (https://github.com/rhysnewell/aviary; v0.3.3), specifying the ont_HQ flag for long reads. In brief, by supplying both short and long reads for hybrid assembly, Aviary first assembled the nanopore reads with metaFlye^77^ (v2.9), then polished the metaFlye assembly using a series of Racon^78^ (v1.4.3; three rounds), Pilon^79^ (v1.23; one round), and Racon (one round). Next, a subset of high-coverage, polished metaFlye contigs were identified and Illumina reads from the same replicate were mapped to them. Illumina reads that did not map to these contigs were assembled using metaSPAdes^80^ (v3.15.3) together with the nanopore reads. Finally, the hybrid-assembled contigs were collated with the high coverage polished metaFlye contigs to produce the final assembly. For the remaining 21 sites not chosen for Nanopore sequencing, a single replicate was assembled using the Aviary pipeline which used metaSPAdes for Illumina-only assembly.

### Prokaryote genome binning and analysis

Assemblies were binned using the Aviary pipeline’s recover workflow, which first mapped reads to each assembly using Minimap2^81^ (v2.18). For differential coverage binning, three sites were chosen for mapping to each assembly based on geographical proximity and all nanopore and Illumina reads for all four replicates from each site were used (Supplementary Table 22). Aviary recover then ran binning programs MetaBAT^82^, MetaBAT 2 (ref. ^83^) (v2.15), MaxBin 2.0 (ref. ^84^) (v2.2.7), Rosella (https://github.com/rhysnewell/rosella; v0.3.3), CONCOCT^85^ (v1.1.0) and Vamb^86^ (v4.0.0) and included a bin refinement step through Rosella to further refine bins based on kmer frequency and coverage. Finally, Das Tool^87^ (v1.1.2) was used to collate a representative set of bins from across the different binning tools. pMAGs were retained if they passed a quality threshold ≥50 (quality = completeness − 3 × contamination) using either CheckM1 (ref. ^88^) (v1.2.1) or CheckM2 (ref. ^89^) (v1.0.2), such that pMAGs of lower completeness were allowed lower contamination scores for inclusion. Taxonomic classification of the pMAGs was performed using the Genome Taxonomy Database Toolkit^90^ (v2.3.0) with database release R214. Dereplication of MAGs for mapping, and for assessment of presence within previously published databases and data sets including the OMD^4^ and Australian National Reference Stations^91^, was carried out using CoverM^92^ cluster (v0.6) at 95% ANI. Relative abundance was calculated by mapping the Illumina reads to the dereplicated set of reference pMAGs using CoverM genome, requiring 95% identity and 75% read length alignment to consider a read mapped.

### Benchmarking hybrid assembly MAG binning

To directly benchmark the effect of including long nanopore data on improving MAG recovery, we undertook separate re-assembly and binning of eight replicates spanning sites from north to south of the GBR. To remove the effect of sequencing depth, we compared two treatments: (1) Illumina-only assembly of 30 Gbp of sequence data using metaSPAdes; and (2) a separate assembly using a subset of 15 Gbp of Illumina reads combined with 15 Gbp of nanopore reads as described above for hybrid assembly. The resulting 16 assemblies were binned with Aviary recover to obtain pMAGs as described above. Genomic diversity of the recovered pMAGs was estimated with inStrain^24^ (v1.7.1) by mapping Illumina reads to their respective metagenome assemblies using Bowtie 2 (ref. ^93^) (v2.4.5) followed by CoverM filter to select mapped reads with at least 95% identity and 75% read alignment.

### Microbial community composition and function

Read mapping counts to the 95% ANI dereplicated set of pMAGs and relative abundances generated using CoverM genome described above were used to visualize community composition and clustering through principal component analysis ordinations in R (ref. ^94^) (v4.4.1) using the vegan^95^ package (v2.6.8). Relative abundances were summarized to genus and other higher taxonomic ranks using phyloseq^96^ (v1.48.0). Associations between community composition, GBR sectors, and reef zoning were assessed using PERMANOVA implemented in vegan, while associations between pMAGs with NTMR and Fished reefs were assessed using generalized linear models implemented in MaAsLin 3 (ref. ^97^) (v0.99.4) controlling for variability across sectors and Benjamini–Hochberg false discovery rate correction for multiple comparisons. Figures showing maps of the GBR and zoning boundaries (for example, Fig. 1) were drawn in R using Geographic Information System data available in the gisaimsr (v0.0.1) R package (https://open-aims.github.io/gisaimsr/index.html).

To assess functional potential of microbial communities in IN and TO sectors, genes were first predicted from IN and TO metagenome assemblies using Pyrodigal^98^ (v3.7.0) followed by clustering at 95% identity using MMseqs2 (ref. ^99^) (v13.45111) to create a non-redundant gene catalogue. Trimmed sequence reads from each IN and TO metagenome were then mapped to this catalogue using CoverM make and read mapping counts subsequently calculated from the output bam files using Samtools^100^ (v1.21). Representative sequences in the non-redundant catalogue were assigned to Kyoto Encyclopedia of Genes and Genomes Orthology using KofamScan^101^ (v1.3.0). Differential abundance statistics on the read mapping counts from CoverM and Samtools were generated using DESeq2 (ref. ^102^) (v1.44) in R.

### Bioindicators of reef zoning

To identify bioindicators of NTMR and Fished reefs, we first performed feature selection using MINT sPLS-DA^103,104^ implemented in mixOmics^105^ (v6.26.0) to account for sectors on the GBR, using centred log ratio-transformed abundances of the 95% ANI dereplicated set of 876 pMAGs, 362,802 vOTUs and 1,136,487 eukaryote contigs (see ‘Final GBR-MGD database compilation’). Random forest (ranger R package v0.17.0) was then used on the selected features to assess accuracy in predicting reef protection zones (average accuracy over 50 runs), using leave one group out cross validation to account for sectors on the GBR. This analysis was then repeated on pMAGs alone with the first MINT sPLS-DA component consisting of the top 60 selected indicator taxa. Differences in average genome size and GC content between indicators of NTMRs and Fished reefs were visualized as box plots using ggplot2 (v3.5.1), and the variation in genome size and GC content was compared between indicators of NTMRs and fished reefs with pairwise Wilcoxon rank-sum tests.

To identify environmental drivers that explained differences in indicator taxa between NTMR and Fished reefs, the 60 indicator pMAGs of reef zoning were correlated with each of the 17 physicochemical variables using MINT sPLS. sPLS^106,107^ models the relationship between multiple predictors (physicochemical variables) and multiple responses (microbial taxa), while MINT^103^ integrates samples from independent subsets to account for confounding effects, such as season and geography. Median values for each physicochemical variable were first calculated per reef site, as the number of replicates differed for molecular (*n* = 4) and water chemistry (*n* = 3) samples. We then correlated centred log ratio-transformed abundances of the 876 dereplicated pMAGs with the 17 physicochemical measurements. Similarity scores (partial correlations) were visualized in ggplot2 (v3.5.1). All analyses were implemented in R. Final composite plots were edited for clarity in Inkscape (v0.92.5).

### Viral, plasmid and eukaryote contigs

The following viral prediction tools were used to identify putative viral contigs from the GBR-MGD metagenomic assemblies: GeNomad^46^ (v1.5.018; --enable-score-calibration --min-score=0.7 --max-score=0.1) with database v1.2, VirSorter2 (ref. ^47^) (v2.2.431; --include-groups “dsDNAphage,NCLDV,RNA,ssDNA,lavidaviridae”, --min-score=0.9) with database v1.2, ViralVerify^108^ (v1.133; -thr 7), VIBRANT^109^ (v1.2.132), PPR-Meta^110^ (v1.134; -t 0.9), and DeepVirFinder^111^ (not versioned; retrieved from GitHub on 8 March 2023; *P* value < 0.05). Putative viral contigs were then input into CheckV^112^ (v1.0.1) with database v1.5 for quality trimming and assessment. Viral predictions were examined using CheckV’s curated marker sets by visualizing the ratio of host:viral markers and the proportion of viral markers for each putative virus prediction to assess whether the contigs predicted by each tool were host- or virus-derived.

In addition to three viral prediction tools that also predicted plasmids (GeNomad, PPRMeta and ViralVerify), two additional plasmid-only prediction tools were used on the metagenomic assemblies, PlasForest^113^ (v1.2), and PlasX^114^ (unversioned; score ≥0.9), which uses annotated gene families generated in anvi’o^115^ (v7.1) using the COG2014 and Pfam v32 databases. Whokaryote^116^ (v1.1.2) and EukRep^117^ (v0.6.6) were used for eukaryotic prediction.

### Manual curation of picoeukaryote genomes

Genomes of two picoeukaryotes from the Mamiellales family, *Bathycoccus prasinos* and *Ostreococcus* sp. clade B, were manually curated after initial blast results of partial eMAGs revealed the presence of potential *Bathycoccus* and *Ostreococcus* long contigs in high abundance as determined by read mapping. Therefore, complete genomes for the sequenced Mamiellales isolates *B. prasinos* RCC1105, *Micromonas commoda* RCC299, *Micromonas pusilla* CCMP1545, *Ostreococcus lucimarinus* CCE9901, *Ostreococcus tauri* and *Ostreococcus* spp. RCC809 were retrieved from the RefSeq database and used as references for competitive mapping of large metagenomic contigs (≥50 kb) with Minimap2 (ref. ^81^) (v2.28) using its genome/assembly alignment mode, allowing up to 20% sequence divergence (--asm20). The majority of contigs were recruited by either *B. prasinos* RCC1105 or *Ostreococcus* spp. RCC809, and were selected for manual curation of two distinct species using the RCC1105 and RCC809 genomes as references. Contigs recruited by *Ostreococcus* spp. RCC809 also showed high recruitment to *O. lucimarinus* CCE9901, which was therefore used as a secondary reference as needed. Contigs were aligned to the corresponding reference using progressiveMauve from the Mauve aligner^118^ (v2015-02-13). In the few cases where a chromosome was recovered in a sample fragmented in 2–4 contigs, Mauve Contig Mover from the Mauve program was used to reorder and link the chromosomal fragments with a linker of 100 Ns. Telomeres were identified at the ends of chromosomal contigs using Tidk^119^ (v0.2.65) to search for telomeric repeat sequence AACCCT, followed by visual inspection to confirm elevated numbers of telomeric repeats at the ends of contigs using Tidk plot.

### Marine *Crassvirales*

vMAGs classified by GeNomad as *Crassvirales* were annotated using Pharokka^120^ (v1.6.1) with additional *Crassvirales* protein profiles provided by Yutin et al.^59^. To verify the GeNomad classifications, we downloaded the set of *Caudoviricetes* TerL sequences compiled by Piedade et al.^62^ and aligned TerL sequences detected in the vMAGs (>400 amino acid residues) to this collection using MAFFT^121^ (v7.508). The output sequence alignment was trimmed with trimAl^122^ (v1.4.rev15; -gt 0.2) and used to infer a bootstrapped phylogenetic tree using IQ-TREE 2 (ref. ^123^) (v2.2.0.3). Separately, we constructed a proteomic tree using the vMAGs (>20 kb) based on genome-wide sequence similarities computed using tBLASTx as implemented in ViPTree^124^ (v4.0). Both trees were visualized and edited for clarity in ITOL^125^ (v7) and Inkscape (v0.92.5). Host prediction was performed on vMAGs using iPHoP^126^ (v1.3.3) with the supplied Aug_2023_pub_rw database including pMAGs dereplicated at 99% ANI (using CoverM) added to this host database.

### Final GBR-MGD database compilation

The GBR-MGD database is composed of prokaryotic MAGs, manually curated Mamiellales genomes, and contigs identified as plasmid, viral, or eukaryotic. A standardized labelling scheme with the structure of IMOS__<TaxaGroup>__SAMPLENAME__CONTIG:<TaxaComponent> was applied to all sequences. TaxaGroup indicates all taxa that were linked to that contig (E = eukaryote, M = plasmid/mobile element, P = prokaryote, V = virus). Contigs marked with only one group were only identified to belong in that group, while contigs marked with multiple letters indicate they were linked to more than one group. For example, a contig marked with a V indicates that the contig was identified as a virus but is not found in another group. PV indicates that a contig is present in a pMAG and predicted to be a virus or provirus, MPV indicates that a contig is found in a pMAG and predicted to be a plasmid and virus. As viruses can be predicted within other taxa (for example, PV contigs), TaxaComponent indicates the taxa represented by a particular sequence as well as the bp position in the case of some viral sequences. For example, a proviral region identified within a prokaryotic bin would be represented by two distinct sequences. The sequence within the prokaryotic bin would be labelled IMOS__PV__SAMPLE__CONTIG:p while its counterpart representing the viral prediction would be labelled IMOS__PV__SAMPLE__CONTIG:v1001-10000. Additionally, contigs are labelled with the prediction tools used to group them into their respective TaxaGroup.

The prokaryotic bins and manually curated *Mamiellales* genomes were dereplicated together using CoverM. Contig predictions were dereplicated separately for each taxon group (for example, V, P, E or PV) using pairwise ANI (95% ANI + 85% alignment fraction) using the anicalc.py and aniclust.py scripts provided by CheckV. Relative abundance of each component of the database was calculated by mapping the Illumina metagenomic reads to each taxon group in the final dereplicated database separately using CoverM Genome, requiring 95% identity and 75% alignment to be considered mapped.

### Reporting summary

Further information on research design is available in the Nature Portfolio Reporting Summary linked to this article.

## Online content

Any methods, additional references, Nature Portfolio reporting summaries, source data, extended data, supplementary information, acknowledgements, peer review information; details of author contributions and competing interests; and statements of data and code availability are available at https://doi.org/10.1038/s41586-026-10778-z.

## Supplementary information

Supplementary Tables Supplementary Tables 1–18 and 20–23

Reporting Summary

Supplementary Table 19 CheckV quality summary including host and viral marker gene counts and quality assessment

Peer Review file

## Data Availability

The GTDB R214 database was downloaded from https://data.gtdb.ecogenomic.org/releases/release214/214.0/. MAGs contained in the OMD v1 were downloaded from https://sunagawalab.ethz.ch/share/microbiomics/ocean/suppl_data/. Metagenomic sequence datasets from the Australian National Reference Stations are available through the Bioplatforms Australia Data Portal (https://data.bioplatforms.com/) by searching for keyword ‘NRS’ and filtering to ‘illumina-shortread’ sequence data type. Genomes of sequenced *Bathycoccus* and *Ostreococcus* are available at RefSeq (https://www.ncbi.nlm.nih.gov/refseq/). *Caudoviricetes* TerL sequences and *Crassvirales* genomes compiled in Piedade et al.^62^ were downloaded from https://dataverse.nioz.nl/dataset.xhtml?persistentId=doi:10.25850/nioz/7b.b.wg. Conserved *Crassvirales* genes from Yutin et al.^59^ were downloaded from https://ftp.ncbi.nlm.nih.gov/pub/yutinn/crassfamily_2020/. The COG2014 database is available at https://ftp.ncbi.nlm.nih.gov/pub/COG/COG2014/data/ and the Pfam32 database is available at https://ftp.ebi.ac.uk/pub/databases/Pfam/releases/Pfam32.0/. Raw sequence data produced in this study are available at the European Nucleotide Archive under project accession PRJEB82623 and secondary accession ERP166296 with project name ‘Great Barrier Reef seawater microbiomes genome database’. All Illumina and Nanopore sample identifiers are provided in Supplementary Table 23. The 5,283 pMAGs, 15 *Ostreococcus* clade B genomes, 5 *Bathycoccus* genomes, 27 *Crassvirales* and group contigs are available at Zenodo.org (https://doi.org/10.5281/zenodo.17109886 (ref. ^127^)).

## Code availability

The R code used for the analyses performed in this study is archived at Zenodo (https://doi.org/10.5281/zenodo.17109886)^127^.
