# Supplementary material for: The planktonic microbiome of the Great Barrier Reef
Source: Nature. 2026 Jul 22;657(8131):426–35. doi: 10.1038/s41586-026-10778-z (PMC13558084; doi:10.1038/s41586-026-10778-z)
Supplement: Supplementary file 1 [file 41586_2026_10778_MOESM2_ESM.pdf]

nature portfolio

Corresponding author(s): David Bourne, Philip Hugenholtz, Yun Kit Yeoh

Last updated by author(s): Jun 1, 2026

Reporting Summary

Nature Portfolio wishes to improve the reproducibility of the work that we publish. This form provides structure for consistency and transparency in reporting. For further information on Nature Portfolio policies, see our Editorial Policies and the Editorial Policy Checklist.

Statistics

For all statistical analyses, confirm that the following items are present in the figure legend, table legend, main text, or Methods section.

n/a	Confirmed	
☐	☒	The exact sample size (*n*) for each experimental group/condition, given as a discrete number and unit of measurement
☐	☒	A statement on whether measurements were taken from distinct samples or whether the same sample was measured repeatedly
☐	☒	The statistical test(s) used AND whether they are one- or two-sided<br>*Only common tests should be described solely by name; describe more complex techniques in the Methods section.*
☐	☒	A description of all covariates tested
☐	☒	A description of any assumptions or corrections, such as tests of normality and adjustment for multiple comparisons
☐	☒	A full description of the statistical parameters including central tendency (e.g. means) or other basic estimates (e.g. regression coefficient) AND variation (e.g. standard deviation) or associated estimates of uncertainty (e.g. confidence intervals)
☐	☒	For null hypothesis testing, the test statistic (e.g. *F*, *t*, *r*) with confidence intervals, effect sizes, degrees of freedom and *P* value noted<br>*Give P values as exact values whenever suitable.*
☒	☐	For Bayesian analysis, information on the choice of priors and Markov chain Monte Carlo settings
☒	☐	For hierarchical and complex designs, identification of the appropriate level for tests and full reporting of outcomes
☒	☐	Estimates of effect sizes (e.g. Cohen's *d*, Pearson's *r*), indicating how they were calculated

Our web collection on statistics for biologists contains articles on many of the points above.

Software and code

Policy information about availability of computer code

Data collection	No software was used.
Data analysis	- MinKNOW v20.06.18 for Nanopore long read sequencing on PromethION 24, Guppy v5.0.16 for Nanopore basecalling<br>- Porechop v0.2.4 for splitting nanopore reads with internal adapters<br>- Aviary pipeline v0.3.3 for metagenome assembly, which invoked metaFlye v2.9 for nanopore long read assembly, racon v1.4.3 and pilon v1.23 for polishing, and metaSPAdes v3.15.3 for hybrid metagenome assembly.<br>- Aviary pipeline v0.3.3 for genome binning, which invoked minimap2 v2.18 for read mapping, and metabat v2.15, maxbin2 v2.2.7, rosella v0.3.3, CONCOCT v1.1.0, VAMB v4.0.0, CheckM v1.2.1, CheckM2 v1.0.2, GTDB-TK v2.3.0, Das Tool v1.1.2, for binning, genome dereplication and taxonomic assignment.<br>- CoverM v0.6 for species dereplication based on average nucleotide identity and read mapping for genome coverage and abundances.<br>- inStrain v1.7.1 and bowtie2 v2.4.5 for hybrid vs Illumina-only genome binning benchmarking<br>- DeepVirFinder (not versioned; retrieved from github 2023-03-08), Genomad v1.5.018, PPR-Meta v1.134, Vibrant v1.2.132, viralverify v1.133, Virsorter2 v2.2.431, CheckV v1.0.1, Plasforest v1.2 and PlasX (unversioned, retrieved from github 20230308) for identification of viral and mobile elements.<br>- Pharokka v1.6.1, MAFFT v7.508, trimAl v1.4rev15, IQTREE2 v2.2.0.3, iPHoP v1.3.3, ViPTree v4.0 and ITOL v7 for phylogenetic inference and visualisation of Crassvirales phylogeny.<br>- Whokaryote v1.1.2, EukRep v0.6.6, anvi'o v7.1 for eukaryotic predictions.<br>- Minimap2 v2.28, Mauve v2015-02-13 and Tidk v0.2.65 for eukaryotic genome alignment and identifying telomeric repeats.<br>- R v4.4.1 with Phyloseq v1.48.0, vegan v2.6.8, ranger v0.17.0, mixomics v6.26.0, maaslin3 v0.99.4, DESeq2 v1.44.0, gisaimsr v0.0.1, ggplot2 v3.5.1 packages for microbial community composition and reef zoning indicator taxa analyses, plotting figures.<br>- Pyrodigal v3.7.0, MMseqs2 v13.45111, samtools v1.21, kofamscan v1.3.0 for community functional analysis using metagenomic sequence

data
- Inkscape v0.92.5 for editing figures in scalable vector graphics (.svg) file format

For manuscripts utilizing custom algorithms or software that are central to the research but not yet described in published literature, software must be made available to editors and reviewers. We strongly encourage code deposition in a community repository (e.g. GitHub). See the Nature Portfolio guidelines for submitting code & software for further information.

Data

Policy information about availability of data

All manuscripts must include a data availability statement. This statement should provide the following information, where applicable:
- Accession codes, unique identifiers, or web links for publicly available datasets
- A description of any restrictions on data availability
- For clinical datasets or third party data, please ensure that the statement adheres to our policy

The GTDB R214 database was downloaded from https://data.gtdb.ecogenomic.org/releases/release214/214.0/. MAGs contained in the Ocean Microbiomics Database (OMD) v1 were downloaded from https://sunagawalab.ethz.ch/share/microbiomics/ocean/suppl_data/. Metagenomic sequence datasets from the Australian National Reference Stations are available through the Bioplatforms Australia Data Portal (https://data.bioplatforms.com/) by searching for keyword “NRS” and filtering to “illumina-shortread” sequence data type. Genomes of sequenced Bathycoccus and Ostreococcus are available at RefSeq https://www.ncbi.nlm.nih.gov/refseq/. Caudoviricetes TerL sequences and Crassvirales genomes compiled in Piedade et al70 were downloaded from https://dataverse.nioz.nl/dataset.xhtml?persistentId=doi:10.25850/nioz/7b.b.wg. Conserved Crassvirales genes from Yutin et al67 were downloaded from https://ftp.ncbi.nlm.nih.gov/pub/yutinn/crassfamily_2020/. The COG2014 database is available at https://ftp.ncbi.nlm.nih.gov/pub/COG/COG2014/data/, Pfam32 database is available at https://ftp.ebi.ac.uk/pub/databases/Pfam/releases/Pfam32.0/. Raw sequence data produced in this study are available at the European Nucleotide Archive under project accession PRJEB82623 and secondary accession ERP166296 with project name “Great Barrier Reef seawater microbiomes genome database”. All Illumina and Nanopore sample identifiers are provided in Supplementary Table 23. The 5,283 pMAGs, 15 Ostreococcus Clade B genomes, 5 Bathycoccus genomes, 27 Crassvirales and group contigs are available at Zenodo.org (https://doi.org/10.5281/zenodo.17109886).

Research involving human participants, their data, or biological material

Policy information about studies with human participants or human data. See also policy information about sex, gender (identity/presentation), and sexual orientation and race, ethnicity and racism.

Reporting on sex and gender	no human participants involved in this study.
Reporting on race, ethnicity, or other socially relevant groupings	no human participants involved in this study.
Population characteristics	no human participants involved in this study.
Recruitment	no human participants involved in this study.
Ethics oversight	no human participants involved in this study.

Note that full information on the approval of the study protocol must also be provided in the manuscript.

Field-specific reporting

Please select the one below that is the best fit for your research. If you are not sure, read the appropriate sections before making your selection.

☐ Life sciences ☐ Behavioural & social sciences ☒ Ecological, evolutionary & environmental sciences

For a reference copy of the document with all sections, see nature.com/documents/nr-reporting-summary-flat.pdf

Ecological, evolutionary & environmental sciences study design

All studies must disclose on these points even when the disclosure is negative.

Study description	Seawater samples were collected from 48 coral reefs across the length of the Great Barrier Reef (GBR) for metagenome sequencing and genome assembly to obtain genome representation of the resident microbial communities. We show that dominant and ubiquitous marine bacteria are often underrepresented by short-read DNA sequencing but can be recovered using Nanopore long read DNA sequencing technology. Analysis of the recovered genomes, including microbes, viruses and eukaryotes demonstrate as yet uncharacterised diversity at the GBR and an ecosystem-wide effect of fisheries management on the microbial communities of surrounding seawater, especially microbes recovered through Nanopore sequencing.
Research sample	At each reef, we collected four 5 L biological replicates of seawater above the reef at about 2 - 10 m depth for a total of 192 samples.
Sampling strategy	Seawater samples were collected using nalgene or Niskin bottles. Each 5 L replicate was filtered through a 5 um filter followed by 0.22 um Sterivex filters to capture microbial cells. Sterivex filters were snap frozen immediately and stored at -75 C.

Data collection	DNA was extracted from the Sterivex filters using a phenol:chloroform protocol at the Australian Institute of Marine Science. Extracted DNA was sent to a sequencing service provider for DNA sequencing.
Timing and spatial scale	Seawater sampling was performed from Nov 2019 - July 2020 at 48 sites across the GBR along Australia's north eastern coast alongside the Australian Institute of Marine Science's GBR Long Term Monitoring Program. These 48 sites span approximately 1600 km of the GBR.
Data exclusions	One of the 192 seawater samples was excluded as wrong chemical was added to Sterivex filter during DNA extraction.
Reproducibility	Four technical seawater replicates were collected at each of the 48 coral reefs. DNA was extracted and sequenced from 191 samples except the one excluded replicate.
Randomization	We used mixomics to account for batch effects associated with geography in analysing microbial taxa indicative of reef management and protection status.
Blinding	Blinding not relevant for the recovery and analysis of microbial genomes.

Did the study involve field work? ☒ Yes ☐ No

Field work, collection and transport

Field conditions	The first three sampling trips occurred in summer and the last trip in winter. No extreme weather events were encountered during the trips.
Location	Seawater samples were collected at 48 coral reefs across the GBR spanning approximately 1600 km. Four 5 L replicates were collected at each reef at 2 - 10 m depth. Figure 1 of the manuscript shows the location of the specific reefs on the GBR.
Access & import/export	Seawater samples were collected under permit G12/35236–1 issued by the Great Barrier Reef Marine Park Authority of the Australian Government. Access was provided through the Australian Institute of Marine Science (AIMS) GBR Long Term Monitoring Program field trips using AIMS research vessels.
Disturbance	Our seawater collection method is unlikely to disturb the reef. Niskin bottles attached to a wire were lowered into the water from the research vessel and brought back to the surface for filtering.

Reporting for specific materials, systems and methods

We require information from authors about some types of materials, experimental systems and methods used in many studies. Here, indicate whether each material, system or method listed is relevant to your study. If you are not sure if a list item applies to your research, read the appropriate section before selecting a response.

Materials & experimental systems

n/a	Involved in the study
☒	☐ Antibodies
☒	☐ Eukaryotic cell lines
☒	☐ Palaeontology and archaeology
☒	☐ Animals and other organisms
☒	☐ Clinical data
☒	☐ Dual use research of concern
☒	☐ Plants

Methods

n/a	Involved in the study
☒	☐ ChIP-seq
☒	☐ Flow cytometry
☒	☐ MRI-based neuroimaging

Plants

Seed stocks	No plants involved in this study.
Novel plant genotypes	No plants involved in this study.
Authentication	No plants involved in this study.
