# Supplementary material for: The planktonic microbiome of the Great Barrier Reef
Source: Nature. 2026 Jul 22;657(8131):426–35. doi: 10.1038/s41586-026-10778-z (PMC13558084; doi:10.1038/s41586-026-10778-z)
Supplement: Supplementary file 2 [file 41586_2026_10778_MOESM4_ESM.pdf]

nature portfolio

Peer Review File

The planktonic microbiome of the Great Barrier Reef

Corresponding Author: Dr Yun Kit Yeoh

Any redactions in this file are there to maintain patient confidentiality, the confidentiality of unpublished data, or to remove third-party material.

This file contains all reviewer reports in order by version, followed by all author rebuttals in order by version.

Version 0:

Reviewer comments:

Referee #1

(Remarks to the Author)
Robbins et al. present the analysis of a large dataset of MAGs obtained from marine samples from the Great Barrier Reef. These MAGs are undoubtedly a great resource: the incorporation of long reads (here ONT) was clearly useful, and the authors have several examples illustrating how this Nanopore sequencing helped with the recovery of some challenging microbes (low-GC, high heterogeneity, or large genomes). The study leverages a unique site (GBR) that has been under-sampled so far, follows all state-of-the-art approaches for metagenomic analysis (with maybe a minor caveat for the description of the viral sequence identification, see below), and is well done and very clearly presented. Most of the manuscript focuses on the improved MAG recovery, and nicely demonstrates how long-read/Nanopore sequencing at scale can improve MAG quality from viruses to microeukaryotes (the latter being especially impressive given their genome size). The main biological analysis reported is a correlation between microbial communities composition and a reef protection status. This is a very nice illustration, although it is not entirely clear to me that this analysis required such a large metagenomic effort: given the magnitude of the signal, I would suspect it would have been picked up even in e.g. 16S amplicon PCR, and the authors do not provide additional analysis that would be uniquely enabled by genome-resolved metagenomics (gene content, functional enrichment/depletion, analysis of other bioindicators such as microeukaryotes or viruses, etc), since GC content and genome size can typically be reasonably extrapolated (to a degree) from 16S amplicons as well.

Outside of my few comments about the virus analysis (below), my main recommendation to the authors would be to be more careful with the wording around capabilities of short-read and long-read metagenomics. The current manuscript make some claims about "inability" of short reads sequencing and some taxa being "not recoverable" which I believe are much too strong considering other published studies, and considering that, while the dataset presented here is relatively large, it is only one type of sample, studied by a single laboratory. I would thus encourage the authors to present the strengths and weaknesses of the different approaches, including their own, more fairly, as right now the current manuscript reads to me as only highlighting flaws in other studies and only highlighting strengths of the authors design and methods.

Major comments:

L. 243 – 256: Most of this paragraph belongs more in the Methods section in my opinion, pipelines for viral sequence prediction have been largely benchmarked at this stage, and are not new enough (in my opinion) to warrant a detailed description as part of the results section.

l. 251: "Surprisingly, we also found that CheckV showed a similar fault, where long contigs were increasingly likely to be designated as complete viral genomes despite having a high ratio of bacterial to viral markers": This is not necessarily surprising, and unfortunately is more a (common) misconception about the tool than anything else. CheckV "contamination" module is only designed to identify short host-derived (contaminating) regions in an otherwise virus contig, and when faced with contigs mostly host-derived, CheckV will not flag them as contamination as there is often no specific region within these contigs that are more "host" than the rest. I would recommend the authors to maybe move this observation to the supplementary materials or the methods, and instead indicate in the results section which tool were used, maybe with a caveat regarding the limitation of combining results from multiple viral sequence prediction tools (see doi 10.1128/msystems.01105-23).

l. 265: “giant phages belonging to the Nucleocytoviricota”: The Nucleocytoviricota phylum currently does not include any phage, and instead is entirely composed of eukaryotic viruses (including protist and algae viruses, likely the ones collected in these samples). Please rephrase.

l. 394: “we suggest that Nanopore long read sequencing should become the standard technology for metagenomic analysis”: I would personally push back against this claim, as I don’t believe the authors provided the data to back it up. First, the authors do not present a comprehensive comparison of sequencing technology, but only show that the addition of ONT to short-read Illumina is useful. Other technologies for long-read sequencing are available and were shown to improve MAG assembly as well (e.g. doi 10.1186/s40168-019-0737-z , 10.1128/msystems.00595-22, or 10.1128/spectrum.03590-23). Second, short-read sequencing seemed to be useful in this study for binning and abundance estimation (which would be expected to be more reliable from short-read Illumina compared to long-read ONT or PacBio). If the authors want to argue that all sequencing for metagenomic should be Nanopore only, they should present what the dataset and analysis would look like when using only these reads. Finally, the authors only study a single sample set from a single type of marine ecosystem with, mostly, a single biological question, while metagenomic is applied to many more sample types and research questions. Given the important cost and input requirement limitations of Nanopore sequencing, and the potential issues that the lower sequencing depth and higher error rate could lead to for other use cases, a much larger benchmarking exercise would have to be presented by the authors to support such a claim of Nanopore becoming the “standard technology for metagenomic analysis”.
I would instead encourage the authors to suggest that Nanopore sequencing “should be considered”, or even “could be included” in all metagenomic analysis for improved MAG recovery.

Minor comments:
L. 33: “Many of the dominant species”: It may be worth mentioning here how these species were identified as dominant (Flow cytometry counts, 16S qPCR, read- or gene-level shotgun metagenomics, etc) ?

L. 35: “these surveys have largely ignored viruses and eukaryotes, with a few notable exceptions”: I think I see what the authors mean, but as currently stated this seems to me quite a harsh statement to make for the many groups that have been working on these topics (viruses and microeukaryotes from shotgun metagenomics) for the last ~ 15-20 years. For instance, the Tara Oceans group published 4 foundational papers about viral diversity in the ocean, based on shotgun metagenomics, which are not cited here (although I appreciate the authors do cite one of the “umbrella” Tara papers). Similarly, several studies already leveraged long-read sequencing to improve the recovery of marine viruses (e.g. doi 10.1101/gr.251686.119, or 10.1186/s40168-022-01340-w) and eukaryotic MAGs have also been actively worked on (e.g. doi 10.1128/mbio.01676-23 or 10.1186/s40168-022-01254-7). I would encourage the authors to rephrase this section, and instead maybe focus on the fact that viruses and eukaryotes are also captured by shotgun metagenomics but can be challenging to assemble.

L. 37: “As are coral reefs”: There was an entire leg of the global Tara expedition dedicated to coral reefs microbiomes, and it is surprising to not see it cited or mentioned at all here (see e.g. doi 10.1371/journal.pbio.3000483, 10.1038/s41597-022-01757-w, 10.1038/s41597-023-02204-0, and a number of other publications).

L. 60: “To generate Australia’s first comprehensive database …”: Given that the notion of “comprehensive” is subjective, I would suggest rephrasing as “To generate a comprehensive database of marine microorganisms from Australia, …” and avoid claims of “first”.

L. 116: “substantial bias in the Illumina reads against pMAGs with low GC content”: Since there is no groundtruth (mock community, or qPCR / ddPCR quantification of individual strains) presented, I believe the authors do not demonstrate how substantial is the bias in Illumina (against low-GC) vs some bias also in ONT (enrichment in low-GC, which has been observed in some cases). For the record, I overall agree with the authors that Nanopore is overall less biased across GC gradients. Still, I would suggest rephrasing to clarify that the direction (or origin) of the bias is not formally, but that low-GC genomes are more represented in the ONT libraries compared to the Illumina libraries.

L. 129: Since other long read technologies exist, and likely provide similar benefits, I would suggest replacing “Nanopore long reads” by “long reads”. Similarly l. 146, with only two methods compared, it is not clear to me that “Nanopore sequencing” was shown as essential, as much as “long read” sequencing

L. 160: “circular pMAGs should [...] have a completeness value of 100%”: It is arguably likely rare, but some microbes are known to encode their genome into multiple large circular DNA molecules/replicons (see e.g. doi 10.1146/annurev.genet.32.1.339, or more recent studies/reviews), which may be worth mentioning here as well ?

L. 183: “TARA” should be “Tara” and in italics (because it is the name of the boat)

L. 203: “lineages that are recalcitrant to recovery using short read sequencing due to high strain heterogeneity”: Since the authors switched in this part to a more biological interpretation of the impact of fisheries on the microbial diversity on and around reefs, I would suggest removing this technical statement and instead say e.g. “lineages displaying high strain heterogeneity …”. In my opinion, the conclusion sentence from l. 223 is sufficient (although again I would caution against claiming “Nanopore” rather than “long read”, and would maybe suggest claiming it to be “important” or “helpful” rather than “necessary”, since there is no demonstration made that the same patterns can not be observed with short-reads only, either shotgun metagenomics and analysis of marker genes, or even 16S PCR).

l. 260: “Caudoviricetes” and all other viral taxa names should all be in italics (per ICTV guidelines).

l. 376: “have not been recoverable”: Given the taxa mentioned in the following sentence, I would suggest rephrasing as “have been challenging to recover”, since MAGs for these taxa have certainly been generated from short-read metagenomes (the sentence l. 378 correctly mentions “underrepresented”).

l. 379: “the inability of short read sequencing”: In the same vein as l. 376, “inability” strikes me as much too strong for what are, ultimately, enrichment/depletion biases.

l. 380: “By contrast, the Nanopore platform addresses both issues by being able to sequence low GC templates and to traverse strain variable regions.” This is correct, however Nanopore sequencing suffers from its own issues (possible small biases towards low GC, relatively high sequencing error rate compared to other sequencing technology, and challenges in recovering rare members of a microbiome due to lower sequencing depth). In my opinion, the authors should include these caveats so that non-expert readers can properly interpret the results presented in the broader context of the marine metagenomic field.

l. 396: “As GC bias is likely inherent to sequencing by synthesis”: This seems like a sweeping and overly broad statement: previous benchmarks like doi 10.1093/gigascience/giaa008 suggested that bias patterns were not just linked to GC content but seemed more complex, and did not affect different sequencing technologies equally (e.g. Illumina vs PacBio). I would recommend toning down this statement.

l. 399: “that could be addressed through Nanopore sequencing”: While I agree this is the case for the GC-related bias, it is not clear to me that Nanopore sequencing specifically (or long-read sequencing in general), would entirely “address” strain heterogeneity. To make such a claim, the authors would need to present datasets with known levels of population diversity and show that Nanopore sequencing is able to recover MAGs for all these levels (which I suspect it may not be able to, especially at lower relative abundance). I would thus separate in these concluding statements the GC bias (for which Nanopore represent a unique approach likely to be less biased than other sequencing technologies) and the strain heterogeneity (for which long-read in general are likely to help, at least partially, but I do not expect any specific strength of Nanopore vs other long-read technologies).

l. 538: “were retrieved”: Please specify from which database these genomes were obtained

l. 588: Please also make available and provide identifiers for all Illumina and Nanopore reads in NCBI SRA (or the equivalent database in an INSDC partner).

Referee #2

(Remarks to the Author)
This study presents the Great Barrier Reef Microbial Genomes Database (GBR-MGD), a microbial genome catalog to date for Australia's Great Barrier Reef (GBR). The authors use a hybrid long-read (Nanopore) and short-read (Illumina) metagenomic approach to recover: >5k prokaryotic MAGs (many circular), 800k viral MAGs, and 20 ~chromosome level eukaryotic MAGs from Mamielalles (Bathycoccus and Ostreococcus). They constructed their metagenomic sequencing efforts to enable the comparison of short-read and hybrid sequencing approaches on the recovery of MAGs, demonstrating that short-read assemblies systematically miss dominant oligotrophic taxa (likely due to high strain heterogeneity and low GC bias). Finally, the authors use the read abundance of pMAGs to develop a model that predicts the environment (Fishing vs. No Take Marine), and highlight the importance of having representative genomes from groups that are often missed by traditional short read sequencing approaches. Notably, the authors have provided excellent documentation of their bioinformatic methods and have developed and used robust pipelines for this work. The paper is well-written and clear, but potentially a bit focused on some of the more technical/performance details of their work (e.g. benchmarking of MAG assembly and viral estimation).

This work represents (to this reviewer’s knowledge) one of the first larger-scale applications of hybrid long-read, short-read metagenomic sequencing to the marine environment (there are a few other examples in other marine regions as well as in other systems e.g. hot springs and human gut). The authors report that ~47% of their reads were recruited to the MAG database that they developed, which is a surprisingly high mapping rate relative to prior studies and is likely related to the recovery of often missed high abundance taxa. This finding has particular relevance to on-going large-scale oceanographic survey programs, some of which are working towards direct mapping of reads against genomic/MAG databases. I do wonder what the mapping rates would have looked like if the authors were to have mapped their data directly to the OMD database. This might be a good analysis to include in the supplement of this paper as it speaks to the potential merits of recovering MAGs specific to a region vs. generating and maintaining a curated set of high-quality MAGs/SAGs/genomes.

The data and methodology used in this study were both robust—with technical replicate metagenomic samples taken across the surveyed sites, the inclusion of the deeply sampled illumina replicate to facilitate nanopore polishing and assembly and well documented bioinformatic analyses. The inclusion of the technical benchmarking comparison between illumina and hybrid sequencing is particularly original and important for the environmental bioinformatics/microbiology community but will likely be less interesting to a broader audience.

My primary critique of the paper is that the vignette examples provided (the biomarkers of reef status, recovery of telomere to telomere picoeuk genomes, and viral MAGs) were not particularly deeply discussed and felt more like use-cases rather than

particularly exciting discoveries made with this dataset. That being said, the paper itself highlights an important new way forward for environmental metagenomics and does an excellent job of benchmarking the approach and highlighting the ecological importance of incorporating long-read data into sampling in the marine system.

Minor comments.

Line 126: Can you add any statistics to back up this statement?

Figure 2: In panel B the y-axis is “Nucleotide diversity (InStrain)” it would be helpful to describe somewhere what this value means. In the text you describe it as a measure of SNP frequency – but it would be helpful to understand what this value actually means.

Figure S2 appears to be a screen shot of an activities guide from a governmental website. Attribution for the figure should be included somewhere (it is possible I just am not finding it in the current text). Also the hand/finger that is caught in the screen grab perhaps should be removed.

Figure S5/S6. Add x and y-axis labels. It isn’t immediately clear what is on the y-axis in particular.

Referee #3

(Remarks to the Author)
This study presents the Great Barrier Reef Microbial Genomes Database (GBR-MGD), a comprehensive genomic inventory of prokaryotes, eukaryotes, and viruses from seawater samples across 48 sites of the Great Barrier Reef (GBR). Using a hybrid approach that combines Nanopore long reads and Illumina short reads, the team generated 5,283 prokaryotic genomes (pMAGs), 20 chromosome-level picoeukaryote genomes, and over 800,000 viral genomes. The authors demonstrate that Nanopore long reads are essential for recovering low-GC and highly strain-heterogeneous taxa (such as Pelagibacter and Prochlorococcus), which are underrepresented in global databases. They also show shifts in microbial communities between fished and no-take marine reserves (NTMRs), identifying microbial indicators linked to reef management status.

Outcomes I would summarize as follows:
- Creation of GBR-MGD: 5,283 prokaryotic genomes, 20 picoeukaryote, 808,585 viral genomes.
- Improved genome recovery of key marine taxa due to hybrid sequencing.
- Identification of underrepresented dominant microbial groups in marine ecosystems.
- Discovery of a novel clade of marine Crassvirales.
- First chromosome-level genomes for key GBR picoeukaryotes (Bathycoccus, Ostreococcus).
- Demonstration that microbial community composition in seawater reflects reef management zones, with potential use as bioindicators.

Conceptual advance:
While the study provides an impressive genomic resource, its primary contribution is technical: improving genome recovery with hybrid sequencing and presenting a large dataset. The conceptual insights into reef microbial ecology remain limited. The finding that microbial communities differ between fished and protected zones is interesting but not deeply mechanistically explored (e.g., specific nutrient fluxes, microbial functional pathways, host interactions).

Mechanistic insight:
The paper identifies community shifts but stops short of elucidating causal mechanisms. A few examples:
- The observation that certain low-GC streamlined taxa are more abundant in NTMRs is only correlated to nutrient levels, without detailed biogeochemical or ecological context.
- The potential functional consequences of viral and picoeukaryotic diversity are not addressed.
- The role of Crassvirales in GBR ecosystems remains speculative.

Of minor concern, but worthwhile pointing out; currently, GBR-MGD is deposited as a sequencing project at ENA (PRJEB82623), limiting its usability to other researchers. The resource is not accessible via a web platform or integrated with existing tools such as mOTUs, IMG/M, or similar frameworks.
To increase its value to the community, the database should be hosted on an interactive web platform with search, download, and visualization tools, or integrated into global efforts like the Ocean Microbiomics Database.

Version 1:

Reviewer comments:

Referee #1

(Remarks to the Author)
I thank the authors for addressing all reviewer comments, and have no further suggestions or concerns at this point.

Referee #2

(Remarks to the Author)
The authors have sufficiently addressed all my previous comments in their revision of the manuscript. From looking over the other reviews, it is obvious to me that they took care to rigorously address reviewer comments. In particular, I appreciated the authors taking the time to map their data to the OMD, as it adds a nice point of comparison with regards to the utility and completeness of the GBR-MGD compared to other large-scale genome compendiums. The key findings, including the under-recovery of low-GC, high-strain-heterogeneity taxa by Illumina-only methods, the recovery of chromosome-level eukaryotic genomes from Bathycoccus and Ostreococcus, the discovery of a distinct high-GC marine Crassvirales clade are compelling example use cases for the hybrid sequencing method. I appreciate the authors' stated limitations regarding space limitations for deeper biological interpretation. The results still read as a sequence of individual vignettes which may benefit from some integrative commentary to link these findings conceptually without a huge amount of expansion of the text (but this is perhaps verging more into a stylistic quibble). I would also note that the tone of the presentation of the work has been balanced in this revision. The manuscript situates itself within existing literature (through response to other reviewers' comments) with reference to other studies on eukaryotic and viral metagenomics. The manuscript has been significantly improved in revision and represents a solid and meaningful contribution to the field of environmental metagenomics/microbiology.

Referee #3

(Remarks to the Author)
I have commented on all aspects (key results, originality, data, statistics, etc) in my original review; besides, some addressal of text revisions I cannot see additional analyses or any deep reflection on my comments from the first round of reviews. The authors do state "we focused the manuscript on the construction of the GBR-MGD and associated technical advances. We agree with the reviewer that further investigation of observed community shifts will identify causal mechanisms (...). We are preparing a subsequent manuscript that focuses on the implicated taxa."

Version 2:

Reviewer comments:

Referee #1

(Remarks to the Author)
The authors addressed all my points in the previous round of revision.

Open Access This Peer Review File is licensed under a Creative Commons Attribution 4.0 International License, which permits use, sharing, adaptation, distribution and reproduction in any medium or format, as long as you give appropriate credit to the original author(s) and the source, provide a link to the Creative Commons license, and indicate if changes were made.
In cases where reviewers are anonymous, credit should be given to 'Anonymous Referee' and the source.
The images or other third party material in this Peer Review File are included in the article's Creative Commons license, unless indicated otherwise in a credit line to the material. If material is not included in the article's Creative Commons license and your intended use is not permitted by statutory regulation or exceeds the permitted use, you will need to obtain

permission directly from the copyright holder.
To view a copy of this license, visit https://creativecommons.org/licenses/by/4.0/

Referees' comments

Referee comments in italics, responses in regular text; line numbers refer to main text document with tracked changes shown.

Referee #1 (Remarks to the Author):

Robbins et al. present the analysis of a large dataset of MAGs obtained from marine samples from the Great Barrier Reef. These MAGs are undoubtedly a great resource: the incorporation of long reads (here ONT) was clearly useful, and the authors have several examples illustrating how this Nanopore sequencing helped with the recovery of some challenging microbes (low-GC, high heterogeneity, or large genomes). The study leverages a unique site (GBR) that has been under-sampled so far, follows all state-of-the-art approaches for metagenomic analysis (with maybe a minor caveat for the description of the viral sequence identification, see below), and is well done and very clearly presented. Most of the manuscript focuses on the improved MAG recovery, and nicely demonstrates how long-read/Nanopore sequencing at scale can improve MAG quality from viruses to microeukaryotes (the latter being especially impressive given their genome size). The main biological analysis reported is a correlation between microbial communities composition and a reef protection status. This is a very nice illustration, although it is not entirely clear to me that this analysis required such a large metagenomic effort: given the magnitude of the signal, I would suspect it would have been picked up even in e.g. 16S amplicon PCR, and the authors do not provide additional analysis that would be uniquely enabled by genome-resolved metagenomics (gene content, functional enrichment/depletion, analysis of other bioindicators such as microeukaryotes or viruses, etc), since GC content and genome size can typically be reasonably extrapolated (to a degree) from 16S amplicons as well.

We appreciate the reviewer's encouraging words regarding the GBR-MGD being an important and useful resource that demonstrates how long reads can markedly improve genomic recovery of dominant taxa, and also for recognizing that Australian oceans and reefs have been underrepresented to date.

We thank the reviewer for the suggestion of using 16S amplicons to predict reef protection status. As we do not have 16S data for these samples, we instead tested the virus, eukaryote and prokaryote fractions collectively and found that they predicted reef protection with 75% accuracy, compared to the 72% achieved with prokaryotes alone, i.e. the standard targets of 16S PCR (added to line 230). This suggests that metagenomes capturing all biodiversity are better at capturing reef states compared to targeted profiles; nevertheless, we concur that 16S amplicons are likely more pragmatic in cost, turnover time for applications related to reef monitoring and health assessment, and thus should be tested on the GBR. We have also toned down the statement at line 268 by removing the sentence "Nanopore sequencing was necessary to identify these patterns".

We agree with the reviewer's comment that further investigation of taxa, gene content and functional enrichment implicated in reef protection is warranted, but feel that such an analysis cannot be accommodated in this manuscript due to length constraints. We are preparing a subsequent manuscript that focuses on implicated taxa.

Outside of my few comments about the virus analysis (below), my main recommendation to the authors would be to be more careful with the wording around capabilities of short-read and long-read metagenomics. The current manuscript make some claims about "inability" of short reads sequencing and some taxa being "not recoverable" which I believe are much too strong considering other published studies, and considering that, while the dataset presented here is relatively large, it is only one type of sample, studied by a single laboratory. I would thus encourage the authors to present the strengths and weaknesses of the different approaches, including their own, more fairly, as right now the current manuscript reads to me as only highlighting flaws in other studies and only highlighting strengths of the authors design and methods.

We thank the reviewer for this more balanced perspective and have toned down our statements as detailed below, and refer to these microbes as "recalcitrant to recovery" or "difficult-to-recover" instead of "not recoverable".

Line 53, originally: "We show that there are several prokaryote taxa from which metagenome-assembled genomes (MAGs) cannot be recovered with Illumina short reads alone due to a combination of high strain heterogeneity and a bias against low GC taxa"

Amended to: "We show that several prokaryote taxa <u>are recalcitrant to</u> recovery with Illumina short reads alone due to a combination of high strain heterogeneity and a bias against low GC taxa"

Line 135, originally: "In addition to high nucleotide diversity, this would account for the inability of Illumina-only assemblies to recover pMAGs from difficult taxa, as all have GC contents below 40%."

Amended to: "In addition to high nucleotide diversity, this would account for the <u>limitations</u> of Illumina-only assemblies in recovering pMAGs from difficult taxa, as all have GC contents below 40%."

Line 141, originally: "The problem reaches a critical threshold above 0.023 nucleotide diversity and <40% GC, where no short read pMAGs could be recovered despite many difficult taxa showing values in this range and being readily recovered in hybrid pMAGs (**Fig. 2B**)."

Amended to: “The problem reaches a critical threshold above 0.02 nucleotide diversity and <40% GC, where no pMAGs could be recovered from the short read assemblies despite many difficult taxa in this range being readily recovered in hybrid assemblies (56 hybrid vs zero Illumina pMAGs) (**Fig. 2B**).”

Line 145, originally: “In summary, we show that assemblies incorporating Nanopore long reads are key to recovery of dominant and ubiquitous marine lineages missed by short read-only assemblies, not only because long reads can overcome elevated strain heterogeneity but because of GC bias inherent to sequencing-by-synthesis platforms.”

Amended to: “In summary, we show that assemblies incorporating long reads substantially enhance the recovery of dominant and ubiquitous marine lineages often missed by short read-only assemblies, not only because long reads can overcome elevated strain heterogeneity but because of GC bias inherent to Illumina sequencing.”

Line 167, originally: “Having established that Nanopore sequencing is essential for representation of cosmopolitan marine lineages, we extended our efforts to additional samples to produce the Great Barrier Reef Microbial Genomes Database, or GBR-MGD.”

Amended to: “Having established that Nanopore sequencing substantially improves representation of cosmopolitan marine lineages, we extended our efforts to additional samples to produce the Great Barrier Reef Microbial Genomes Database, or GBR-MGD.”

Line 443, originally: “Metagenomics has greatly enabled the study of microbial ecosystems by bypassing the cultivation bottleneck. However, marine ecosystems are unusual in that the genomes of many dominant microbial populations have not been recoverable through standard short read metagenomics.”

Amended to: “Metagenomics has greatly enabled the study of microbial ecosystems by bypassing the cultivation bottleneck. However, marine ecosystems are unusual in that the genomes of many dominant microbial populations are conspicuously underrepresented in short-read MAG datasets.”

Line 469, originally: “Moving forward, we suggest that Nanopore long read sequencing should become the standard technology for metagenomic analysis of marine microbial communities, as many key populations are only accessible via this platform. As GC bias is likely inherent to sequencing by synthesis technologies, and strain heterogeneity is not limited to marine environments, we predict that the sequencing limitations observed in this study will affect

metagenomic analyses of other ecosystems that could be addressed through Nanopore sequencing."

Amended to: "Moving forward, we suggest that long read sequencing should become a standard part of metagenomic analyses of marine microbial communities, as many key populations are poorly accessible via short read sequencing alone. As low-GC taxa and strain heterogeneity are not limited to marine environments, we predict that the sequencing limitations observed in this study will affect metagenomic analyses of other ecosystems that could be addressed through the incorporation of long reads".

Major comments:

L. 243 – 256: Most of this paragraph belongs more in the Methods section in my opinion, pipelines for viral sequence prediction have been largely benchmarked at this stage, and are not new enough (in my opinion) to warrant a detailed description as part of the results section.

We appreciate the reviewer's comment but suggest that a brief overview of the viral tools used here is necessary because their performance has not been evaluated on long read assemblies, and we show that this significantly impacts their performance. For example, DeepVirFinder is a widely cited viral identification tool with close to 600 citations (DOI 10.1007/s40484-019-0187-4) but performs poorly on long read assemblies. To be clearer, we edited the text to emphasize that here we apply these tools to long read assemblies:

Line 290, originally: "Viral detection tools perform poorly on highly fragmented assemblies with shorter contigs, and consequently produce variable results. Therefore, we first tested six widely used metagenomic viral prediction tools to identify viruses in the GBR-MGD; GeNomad, Virsorter2, ViralVerify, VIBRANT, PPR-Meta, and DeepVirFinder, using CheckV to assess the number of complete viruses predicted by each tool."

Amended to: "Current state of the art viral detection tools have almost exclusively been benchmarked on short-read assemblies, and are known to be sensitive to contig length. To assess their use on long read assemblies, we first tested six widely used metagenomic viral prediction tools to identify viruses in the GBR-MGD; GeNomad, Virsorter2, ViralVerify, VIBRANT, PPR-Meta, and DeepVirFinder, using CheckV to assess the number of complete viruses predicted by each tool."

l. 251: "Surprisingly, we also found that CheckV showed a similar fault, where long contigs were increasingly likely to be designated as complete viral genomes despite having a high ratio of

bacterial to viral markers": This is not necessarily surprising, and unfortunately is more a (common) misconception about the tool than anything else. CheckV "contamination" module is only designed to identify short host-derived (contaminating) regions in an otherwise virus contig, and when faced with contigs mostly host-derived, CheckV will not flag them as contamination as there is often no specific region within these contigs that are more "host" than the rest. I would recommend the authors to maybe move this observation to the supplementary materials or the methods, and instead indicate in the results section which tool were used, maybe with a caveat regarding the limitation of combining results from multiple viral sequence prediction tools (see doi 10.1128/msystems.01105-23).

We agree with the reviewer and have removed this sentence. However, we believe it is worth highlighting to users a scenario created by the use of long reads in which DeepVirFinder classifies a large percentage of non-viral contigs as viruses, which users would then pass to CheckV for quality assessment. CheckV will then frequently classify them as "high quality viruses." An expert user may undertake their own benchmarking and catch this behaviour, but this mistake will be undetected for many CheckV users and could result in extensive misidentification of viral auxiliary metabolic genes from prokaryote sequences. Therefore, we have amended the wording of this section as follows:

Line 299, originally: "DeepVirFinder showed substantially more false positives than any other tool, with the proportion of bacterial marker genes among putative viral contigs increasing with contig length, indicating clear bacterial origin (**Fig. S5 and S6**). Surprisingly, we also found that CheckV showed a similar fault, where long contigs were increasingly likely to be designated as complete viral genomes despite having a high ratio of bacterial to viral markers (**Fig. S5 and S6**).".

Has been amended to: "DeepVirFinder showed substantially more false positives than the other tools tested, with the proportion of bacterial marker genes among putative viral contigs increasing with contig length, indicating bacterial origin (**Fig. S5 and S6**). <u>When these bacterial contigs were assessed by CheckV, longer contigs were increasingly likely to be designated as complete viral genomes, despite having a high ratio of bacterial to viral markers (**Fig. S5 and S6**). We note that CheckV is not designed to identify viral vs non-viral sequences, but highlight this result because it is common practice to filter assemblies based on CheckV quality scores that could result in extensive misidentification of viral auxiliary metabolic genes if DeepVirFinder is applied to long read assemblies</u>."

Additionally, we added a sentence in the methods (line 669) to highlight that the sequence headers of the virus and other taxa group contigs indicate the identification tools (or single tool) that predict its taxa group. This gives users the flexibility to choose whether to work with contigs predicted by a single tool or a combination of identification tools.

l. 265: "giant phages belonging to the Nucleocytoviricota": The Nucleocytoviricota phylum currently does not include any phage, and instead is entirely composed of eukaryotic viruses (including protist and algae viruses, likely the ones collected in these samples). Please rephrase.

Agreed. We have changed "phages" to "viruses" (line 331).

l. 394: "we suggest that Nanopore long read sequencing should become the standard technology for metagenomic analysis": I would personally push back against this claim, as I don't believe the authors provided the data to back it up. First, the authors do not present a comprehensive comparison of sequencing technology, but only show that the addition of ONT to short-read Illumina is useful. Other technologies for long-read sequencing are available and were shown to improve MAG assembly as well (e.g. doi 10.1186/s40168-019-0737-z , 10.1128/msystems.00595-22, or 10.1128/spectrum.03590-23). Second, short-read sequencing seemed to be useful in this study for binning and abundance estimation (which would be expected to be more reliable from short-read Illumina compared to long-read ONT or PacBio). If the authors want to argue that all sequencing for metagenomic should be Nanopore only, they should present what the dataset and analysis would look like when using only these reads. Finally, the authors only study a single sample set from a single type of marine ecosystem with, mostly, a single biological question, while metagenomic is applied to many more sample types and research questions. Given the important cost and input requirement limitations of Nanopore sequencing, and the potential issues that the lower sequencing depth and higher error rate could lead to for other use cases, a much larger benchmarking exercise would have to be presented by the authors to support such a claim of Nanopore becoming the "standard technology for metagenomic analysis".

I would instead encourage the authors to suggest that Nanopore sequencing "should be considered", or even "could be included" in all metagenomic analysis for improved MAG recovery.

We acknowledge the reviewer's points regarding overstating the capabilities of ONT and have amended the text as detailed above in response to a related comment. In particular, the sentence highlighted by the reviewer in this comment has been edited:

Line 469, originally: "Moving forward, we suggest that Nanopore long read sequencing should become the standard technology for metagenomic analysis of marine microbial communities, as many key populations are only accessible via this platform. As GC bias is likely inherent to sequencing by synthesis technologies, and strain heterogeneity is not limited to marine environments, we predict that the sequencing limitations observed in this study will affect metagenomic analyses of other ecosystems that could be addressed through Nanopore sequencing."

Amended to: "Moving forward, we suggest that long read sequencing should become a standard part of metagenomic analyses of marine microbial communities, as many key populations are

poorly accessible via short read sequencing alone. As low-GC taxa and strain heterogeneity are not limited to marine environments, we predict that the sequencing limitations observed in this study will affect metagenomic analyses of other ecosystems that could be addressed through the incorporation of long reads".

Minor comments:

L. 33: "Many of the dominant species": It may be worth mentioning here how these species were identified as dominant (Flow cytometry counts, 16S qPCR, read- or gene-level shotgun metagenomics, etc) ?

We edited the sentence at line 37 to indicate that the numerical abundances of these dominant species were measured through flow cytometry counts and metagenomic read mapping to metagenome assembled genomes as per the studies cited here (reference list 8 and 9).

The edited sentence is: "Amongst the prokaryotic inventories, however, many of the dominant species are conspicuously underrepresented <u>as evidenced by flow cytometry cell counts and metagenomic read mapping</u>, including *Pelagibacter*, *Prochlorococcus*, SAR86, and others".

L. 35: "these surveys have largely ignored viruses and eukaryotes, with a few notable exceptions": I think I see what the authors mean, but as currently stated this seems to me quite a harsh statement to make for the many groups that have been working on these topics (viruses and microeukaryotes from shotgun metagenomics) for the last ~ 15-20 years. For instance, the Tara Oceans group published 4 foundational papers about viral diversity in the ocean, based on shotgun metagenomics, which are not cited here (although I appreciate the authors do cite one of the "umbrella" Tara papers). Similarly, several studies already leveraged long-read sequencing to improve the recovery of marine viruses (e.g. doi 10.1101/gr.251686.119, or 10.1186/s40168-022-01340-w) and eukaryotic MAGs have also been actively worked on (e.g. doi 10.1128/mbio.01676-23 or 10.1186/s40168-022-01254-7). I would encourage the authors to rephrase this section, and instead maybe focus on the fact that viruses and eukaryotes are also captured by shotgun metagenomics but can be challenging to assemble.

We agree with the reviewer's comment and have rephrased as follows:

Line 40, originally: "Further, these surveys have largely ignored viruses and eukaryotes, with a few notable exceptions."

Amended to: "Further, the viral and eukaryote components of metagenomic surveys are less often included, with a few notable exceptions."

We have added the references suggested by the reviewer describing virus and eukaryotic metagenomics in marine ecosystems– Roux et al., (2016), Gregory et al (2019), Duncan et al., (2022), Alexander et al., (2023).

L. 37: "As are coral reefs": There was an entire leg of the global Tara expedition dedicated to coral reefs microbiomes, and it is surprising to not see it cited or mentioned at all here (see e.g. doi 10.1371/journal.pbio.3000483, 10.1038/s41597-022-01757-w, 10.1038/s41597-023-02204-0, *and a number of other publications).*

We clarify that we are referring specifically to Australia's coral reefs as follows:

Line 44, originally: "Australia's oceans are also mostly absent in global marine genome surveys, as are coral reefs, which are hotspots for biodiversity."

Amended to: "Australia's oceans are also mostly absent in global marine genome surveys, as are its coral reefs, which are hotspots for biodiversity."

We have also added references highlighted by the reviewer to acknowledge coral reef metagenomic surveys conducted by *Tara* Pacific.

L. 60: "To generate Australia's first comprehensive database …": Given that the notion of "comprehensive" is subjective, I would suggest rephrasing as "To generate a comprehensive database of marine microorganisms from Australia, …" and avoid claims of "first".

We edited the sentence as suggested at line 73. The new sentence is: "To generate a comprehensive database of GBR marine microorganisms".

L. 116: "substantial bias in the Illumina reads against pMAGs with low GC content": Since there is no groundtruth (mock community, or qPCR / ddPCR quantification of individual strains) presented, I believe the authors do not demonstrate how substantial is the bias in Illumina (against low-GC) vs some bias also in ONT (enrichment in low-GC, which has been observed in some cases). For the record, I overall agree with the authors that Nanopore is overall less biased across GC gradients. Still, I would suggest rephrasing to clarify that the direction (or origin) of the bias is not formally, but that low-GC genomes are more represented in the ONT libraries compared to the Illumina libraries.

We edited the sentence as suggested to avoid directly ascribing the bias to Illumina or Nanopore given the lack of ground truthing, and cite literature indicating that Illumina sequencing but not Nanopore tends to underrepresent low GC sequences (Browne et al., 2020 DOI 10.1093/gigascience/giaa008).

Line 130, originally**:** "a clear and substantial bias in the Illumina reads against pMAGs with low GC content"

Has been amended to: "we found that low GC pMAGs were better represented in Nanopore than Illumina data".

L. 129: Since other long read technologies exist, and likely provide similar benefits, I would suggest replacing "Nanopore long reads" by "long reads". Similarly l. 146, with only two methods compared, it is not clear to me that "Nanopore sequencing" was shown as essential, as much as "long read" sequencing

We replaced "Nanopore long reads" with "long reads" at line 145 (originally line 129). At line 167 (originally line 146), we edited the sentence to indicate that Nanopore sequencing improved representation of the marine lineages instead of the original text saying "Nanopore sequencing is essential".

Line 167, originally: "Having established that Nanopore sequencing is essential for representation of cosmopolitan marine lineages"

Amended to: "Having established that Nanopore sequencing substantially improves representation of cosmopolitan marine lineages".

L. 160: "circular pMAGs should [...] have a completeness value of 100%": It is arguably likely rare, but some microbes are known to encode their genome into multiple large circular DNA molecules/replicons (see e.g. doi 10.1146/annurev.genet.32.1.339, or more recent studies/reviews), which may be worth mentioning here as well ?

We have added a caveat to this sentence regarding multipartite bacterial genomes as suggested (line 181).

L. 183: "TARA" should be "Tara" and in italics (because it is the name of the boat)

We replaced TARA with *Tara* throughout the manuscript.

L. 203: "lineages that are recalcitrant to recovery using short read sequencing due to high strain heterogeneity": Since the authors switched in this part to a more biological interpretation of the impact of fisheries on the microbial diversity on and around reefs, I would suggest removing this technical statement and instead say e.g. "lineages displaying high strain heterogeneity …". In my

opinion, the conclusion sentence from l. 223 is sufficient (although again I would caution against claiming "Nanopore" rather than "long read", and would maybe suggest claiming it to be "important" or "helpful" rather than "necessary", since there is no demonstration made that the same patterns can not be observed with short-reads only, either shotgun metagenomics and analysis of marker genes, or even 16S PCR).

We replaced the part on "lineages that are recalcitrant to recovery using short read sequencing" with "displaying high strain heterogeneity and low GC content" as suggested to simplify the sentence.

Line 245, originally: "all identified in the present study as lineages <u>that are recalcitrant to recovery using short read sequencing due to</u> high strain heterogeneity and low GC content"

Amended to: "all identified in the present study as lineages <u>displaying</u> high strain heterogeneity and low GC content".

Additionally, we edited the concluding sentence in this section (originally line 225) to avoid claiming that long reads were crucial to the observation, instead highlighting that long reads helped deliver the pMAGs implicated in reef protection zoning.

Line 268, originally: "The inability of short-read sequencing to deliver pMAGs from streamlined, low-GC taxa, which are ubiquitous in marine ecosystems, has not previously been appreciated and highlights that Nanopore sequencing was necessary to identify these patterns"

Amended to: "The difficulty in obtaining pMAGs of streamlined, low-GC taxa from short read datasets alone would likely compromise the ability to predict reef protection status using this approach".

l. 260: "Caudoviricetes" and all other viral taxa names should all be in italics (per ICTV guidelines).

We have italicised all occurrences of viral taxa names in the manuscript.

l. 376: "have not been recoverable": Given the taxa mentioned in the following sentence, I would suggest rephrasing as "have been challenging to recover", since MAGs for these taxa have certainly been generated from short-read metagenomes (the sentence l. 378 correctly mentions "underrepresented").

We edited the sentence as suggested.

Line 444, originally: "However, marine ecosystems are unusual in that the genomes of many dominant microbial populations have not been recoverable through standard short read metagenomics"

Amended to: "However, marine ecosystems are unusual in that the genomes of many dominant microbial populations <u>are conspicuously underrepresented</u> in short-read MAG datasets".

l. 379: "the inability of short read sequencing": In the same vein as l. 376, "inability" strikes me as much too strong for what are, ultimately, enrichment/depletion biases.

We replaced the word "inability" with "limitations".

Line 449, originally: "We show that this phenomenon is due to the inability of short read sequencing and assembly to handle the combination of high strain heterogeneity and low GC content of these taxa."

Amended to: "We show that this phenomenon is due to <u>limitations</u> of short read sequencing and assembly to handle the combination of high strain heterogeneity and low GC content of these taxa"

l. 380: "By contrast, the Nanopore platform addresses both issues by being able to sequence low GC templates and to traverse strain variable regions." This is correct, however Nanopore sequencing suffers from its own issues (possible small biases towards low GC, relatively high sequencing error rate compared to other sequencing technology, and challenges in recovering rare members of a microbiome due to lower sequencing depth). In my opinion, the authors should include these caveats so that non-expert readers can properly interpret the results presented in the broader context of the marine metagenomic field.

We added a sentence mentioning the limitations of Nanopore sequencing as suggested at line 453.

The amended text is: "By contrast, the Nanopore platform addresses both issues by being able to sequence low GC templates and to traverse strain variable regions. <u>However, Nanopore sequencing has its own challenges such as higher error rates in the raw reads and lower data output compared to Illumina platforms. Therefore, hybrid sequencing approaches that include long reads will produce the most comprehensive and high quality genome inventory of marine microbial taxa using currently available technologies</u>".

l. 396: "As GC bias is likely inherent to sequencing by synthesis": This seems like a sweeping and overly broad statement: previous benchmarks like doi 10.1093/gigascience/giaa008 suggested that bias patterns were not just linked to GC content but seemed more complex, and did not affect

different sequencing technologies equally (e.g. Illumina vs PacBio). I would recommend toning down this statement.

We concur that since it is not directly addressed here whether other long read sequencing technologies are equally impacted by the low GC marine populations, we thus edited the paragraph beginning to avoid comparison to other long reads technologies that we did not test.

The amended text (line 469): "Moving forward, we suggest that long read sequencing should become a standard part of metagenomic analyses of marine microbial communities, as many key populations are poorly accessible via short read sequencing alone. As low-GC taxa and strain heterogeneity are not limited to marine environments, we predict that the sequencing limitations observed in this study will affect metagenomic analyses of other ecosystems that could be addressed through the incorporation of long reads."

l. 399: "that could be addressed through Nanopore sequencing": While I agree this is the case for the GC-related bias, it is not clear to me that Nanopore sequencing specifically (or long-read sequencing in general), would entirely "address" strain heterogeneity. To make such a claim, the authors would need to present datasets with known levels of population diversity and show that Nanopore sequencing is able to recover MAGs for all these levels (which I suspect it may not be able to, especially at lower relative abundance). I would thus separate in these concluding statements the GC bias (for which Nanopore represent a unique approach likely to be less biased than other sequencing technologies) and the strain heterogeneity (for which long-read in general are likely to help, at least partially, but I do not expect any specific strength of Nanopore vs other long-read technologies).

As part of the changes made to address the reviewer's other comments, the edited paragraph takes a more balanced viewpoint of highlighting "long reads" in general rather than Nanopore alone.

Line 477 (originally line 396-399): "As GC bias is likely inherent to sequencing by synthesis technologies, and strain heterogeneity is not limited to marine environments, we predict that the sequencing limitations observed in this study will affect metagenomic analyses of other ecosystems that could be addressed through Nanopore sequencing."

Has been amended to: "As low-GC taxa and strain heterogeneity are not limited to marine environments, we predict that the sequencing limitations observed in this study will affect metagenomic analyses of other ecosystems that could be addressed through the incorporation of long reads."

l. 538: "were retrieved": Please specify from which database these genomes were obtained

We edited the sentence to specify that these genomes were obtained from RefSeq (line 627).

l. 588: Please also make available and provide identifiers for all Illumina and Nanopore reads in NCBI SRA (or the equivalent database in an INSDC partner).

We edited the data availability statement to indicate that the identifiers are provided in supplementary table S17 (line 681)**.**

Referee #2 (Remarks to the Author):

This study presents the Great Barrier Reef Microbial Genomes Database (GBR-MGD), a microbial genome catalog to date for Australia's Great Barrier Reef (GBR). The authors use a hybrid long-read (Nanopore) and short-read (Illumina) metagenomic approach to recover: >5k prokaryotic MAGs (many circular), 800k viral MAGs, and 20 ~chromosome level eukaryotic MAGs from Mamielalles (Bathycoccus and Ostreococcus). They constructed their metagenomic sequencing efforts to enable the comparison of short-read and hybrid sequencing approaches on the recovery of MAGs, demonstrating that short-read assemblies systematically miss dominant oligotrophic taxa (likely due to high strain heterogeneity and low GC bias). Finally, the authors use the read abundance of pMAGs to develop a model that predicts the environment (Fishing vs. No Take Marine), and highlight the importance of having representative genomes from groups that are often missed by traditional short read sequencing approaches. Notably, the authors have provided excellent documentation of their bioinformatic methods and have developed and used robust pipelines for this work. The paper is well-written and clear, but potentially a bit focused on some of the more technical/performance details of their work (e.g. benchmarking of MAG assembly and viral estimation).

We thank the reviewer for their supportive assessment of the manuscript. We address the comment about focusing on the technical aspects below.

This work represents (to this reviewer's knowledge) one of the first larger-scale applications of hybrid long-read, short-read metagenomic sequencing to the marine environment (there are a few other examples in other marine regions as well as in other systems e.g. hot springs and human gut). The authors report that ~47% of their reads were recruited to the MAG database that they developed, which is a surprisingly high mapping rate relative to prior studies and is likely related to the recovery of often missed high abundance taxa. This finding has particular relevance to on-going large-scale oceanographic survey programs, some of which are working towards direct mapping of reads against genomic/MAG databases. I do wonder what the mapping rates would have looked like if the authors were to have mapped their data directly to the OMD database. This might be a good analysis to include in the supplement of this paper as it speaks to the potential

merits of recovering MAGs specific to a region vs. generating and maintaining a curated set of high-quality MAGs/SAGs/genomes.

We mapped the GBR-MGD Illumina reads to OMD MAGs and SAGs described in Paoli et al. (DOI 10.1038/s41586-022-04862-3). This resulted in an average 40.4±7.7% (maximum 53.7%) of the GBR community represented by OMD MAGs. We have incorporated these findings (line 212) to highlight GBR microbial diversity not yet captured in the OMD.

The data and methodology used in this study were both robust—with technical replicate metagenomic samples taken across the surveyed sites, the inclusion of the deeply sampled illumina replicate to facilitate nanopore polishing and assembly and well documented bioinformatic analyses. The inclusion of the technical benchmarking comparison between illumina and hybrid sequencing is particularly original and important for the environmental bioinformatics/microbiology community but will likely be less interesting to a broader audience.

We appreciate the reviewer's perspective regarding the technical benchmarking aspects of the manuscript but suggest that this section is necessary as it demonstrates the limitations of short read sequencing alone in recovering taxa with low GC and high strain heterogeneity, and how long reads improves genome recovery of these taxa, which are typical of marine microbial communities. It provides reason for the inclusion of nanopore long reads in this study that sets the GBR-MGD apart from other large scale marine metagenomic surveys which use only short reads. We believe that the methods described herein are applicable across diverse ecosystems, which should appeal to a broad audience.

My primary critique of the paper is that the vignette examples provided (the biomarkers of reef status, recovery of telomere to telomere picoeuk genomes, and viral MAGs) were not particularly deeply discussed and felt more like use-cases rather than particularly exciting discoveries made with this dataset. That being said, the paper itself highlights an important new way forward for environmental metagenomics and does an excellent job of benchmarking the approach and highlighting the ecological importance of incorporating long-read data into sampling in the marine system.

We acknowledge the reviewer's comment that further investigation of the GBR-MGD data set is warranted, but feel that such analyses cannot be accommodated in this manuscript due to length constraints. We focus on the methodological advancements that set the MGD apart from existing large marine metagenomic surveys and benchmarking to describe microbial diversity unique to the GBR, thus highlighting strengths of this data set we are certain will drive exciting discoveries that warrant standalone manuscripts. Nevertheless, we thank the reviewer for recognizing the value of this genomic data set and its ecological importance towards better managing the GBR.

Minor comments.

Line 126: Can you add any statistics to back up this statement?

We added the number of pMAGs recovered from hybrid vs short-read only assemblies to support this statement (56 from hybrid assemblies, zero from short reads) (line 144).

Figure 2: In panel B the y-axis is "Nucleotide diversity (InStrain)" it would be helpful to describe somewhere what this value means. In the text you describe it as a measure of SNP frequency – but it would be helpful to understand what this value actually means.

Nucleotide diversity is a measure of genetic diversity in a microbial population implemented in inStrain (DOI 10.1038/s41587-020-00797-0). We amended text at line 119 and the legend of figure 2 to explain this measure.

Figure S2 appears to be a screen shot of an activities guide from a governmental website. Attribution for the figure should be included somewhere (it is possible I just am not finding it in the current text). Also the hand/finger that is caught in the screen grab perhaps should be removed.

We removed this supplemental figure and now provide a reference to the Australian Government Great Barrier Reef (GBR) Marine Park Zoning Plan document that includes descriptions of the zoning categories, and the list of reefs designated within each category. This document contains more in-depth information about the GBR zones than the original screen shot. We now provide a web link to the zoning maps (from which the screen shot was taken) in the legend of Figure S3 which is an adaptation of the zoning maps.

Figure S5/S6. Add x and y-axis labels. It isn't immediately clear what is on the y-axis in particular.

We have added axis labels to figures S5 and S6.

Referee #3 (Remarks to the Author):

This study presents the Great Barrier Reef Microbial Genomes Database (GBR-MGD), a comprehensive genomic inventory of prokaryotes, eukaryotes, and viruses from seawater samples across 48 sites of the Great Barrier Reef (GBR). Using a hybrid approach that combines Nanopore long reads and Illumina short reads, the team generated 5,283 prokaryotic genomes (pMAGs), 20 chromosome-level picoeukaryote genomes, and over 800,000 viral genomes. The authors

demonstrate that Nanopore long reads are essential for recovering low-GC and highly strain-heterogeneous taxa (such as Pelagibacter and Prochlorococcus), which are underrepresented in global databases. They also show shifts in microbial communities between fished and no-take marine reserves (NTMRs), identifying microbial indicators linked to reef management status.

Outcomes I would summarize as follows:

- Creation of GBR-MGD: 5,283 prokaryotic genomes, 20 picoeukaryote, 808,585 viral genomes.

- Improved genome recovery of key marine taxa due to hybrid sequencing.

- Identification of underrepresented dominant microbial groups in marine ecosystems.

- Discovery of a novel clade of marine Crassvirales.

- First chromosome-level genomes for key GBR picoeukaryotes (Bathycoccus, Ostreococcus).

- Demonstration that microbial community composition in seawater reflects reef management zones, with potential use as bioindicators.

Conceptual advance:

While the study provides an impressive genomic resource, its primary contribution is technical: improving genome recovery with hybrid sequencing and presenting a large dataset. The conceptual insights into reef microbial ecology remain limited. The finding that microbial communities differ between fished and protected zones is interesting but not deeply mechanistically explored (e.g., specific nutrient fluxes, microbial functional pathways, host interactions).

Mechanistic insight:

The paper identifies community shifts but stops short of elucidating causal mechanisms. A few examples:

- The observation that certain low-GC streamlined taxa are more abundant in NTMRs is only correlated to nutrient levels, without detailed biogeochemical or ecological context.

- The potential functional consequences of viral and picoeukaryotic diversity are not addressed.

- The role of Crassvirales in GBR ecosystems remains speculative.

We thank the reviewer for their summary of the manuscript. Regarding limited mechanistic insights and conceptual advances, we focused the manuscript on the construction of the GBR-MGD and associated technical advances. We agree with the reviewer that further investigation of observed community shifts will identify causal mechanisms, but feel that such an analysis cannot be accommodated in this manuscript due to length constraints. We are preparing a subsequent manuscript that focuses on the implicated taxa.

Of minor concern, but worthwhile pointing out; currently, GBR-MGD is deposited as a sequencing project at ENA (PRJEB82623), limiting its usability to other researchers. The resource is not accessible via a web platform or integrated with existing tools such as mOTUs, IMG/M, or similar frameworks.

To increase its value to the community, the database should be hosted on an interactive web platform with search, download, and visualization tools, or integrated into global efforts like the Ocean Microbiomics Database.

We are in contact with the developers of the Ocean Microbiomics Database (OMD). The OMD only accepts genomes from published articles as part of their criteria to maintain traceability; the GBR-MGD genomes will be incorporated into the OMD upon publication. Additionally, these genomes will be incorporated into the Genome Taxonomy Database (https://gtdb.ecogenomic.org/) established and maintained by Phil Hugenholtz who is a coauthor on this study.

Editor's comments

More specifically, given the reservations raised by Referee #3, and our own editorial expectations, we would need you to incorporate additional biological insights in the revised version for it to be considered further. For example, this could include changes in the relative abundances of key taxa and functional distinctions among the indicator species. Technical details may be moved to the Supplementary Information, allowing the main text to focus more clearly on the marine microbial communities as potential indicators of reef protection status.

We have incorporated additional biological insights as detailed below.

Response to referees' comments

Line numbers quoted in this response document correspond to the manuscript file with changes tracked.

Referee #1 (Remarks to the Author):

I thank the authors for addressing all reviewer comments, and have no further suggestions or concerns at this point.

We thank Referee 1 for their time and feedback in reviewing the manuscript.

Referee #2 (Remarks to the Author):

The authors have sufficiently addressed all my previous comments in their revision of the manuscript. From looking over the other reviews, it is obvious to me that they took care to rigorously address reviewer comments. In particular, I appreciated the authors taking the time to map their data to the OMD, as it adds a nice point of comparison with regards to the utility and completeness of the GBR-MGD compared to other large-scale genome compendiums. The key findings, including the under-recovery of low-GC, high-strain-heterogeneity taxa by Illumina-only methods, the recovery of chromosome-level eukaryotic genomes from Bathycoccus and Ostreococcus, the discovery of a distinct high-GC marine Crassvirales clade are compelling example use cases for the hybrid sequencing method. I appreciate the authors' stated limitations regarding space limitations for deeper biological interpretation.

We have now included analyses of the microbial community composition and show adaptations at the gene family level to nutrient availability (lines 209-226). We also identify region-specific taxa and functions associated with no-take marine reserves (NTMRs) and fished reefs, highlighting regional differences within the broader enrichment of oligotrophic taxa in NTMRs at the Great Barrier Reef (lines 269-310).

The results still read as a sequence of individual vignettes which may benefit from some integrative commentary to link these findings conceptually without a huge amount of expansion of the text (but this is perhaps verging more into a stylistic quibble).

We edited the discussion (lines 497-516) linking findings facilitated by the GBR-MGD, including prokaryotes, viruses and eukaryotes, with seawater physicochemistry and reef management to highlight biological insight into ecosystem function afforded by DNA data, and how the integration of these information enables diagnostic and adaptive reef monitoring on the GBR and beyond.

I would also note that the tone of the presentation of the work has been balanced in this revision. The manuscript situates itself within existing literature (through response to other reviewers' comments) with reference to other studies on eukaryotic and viral metagenomics. The manuscript has been significantly improved in revision and represents a solid and meaningful contribution to the field of environmental metagenomics/microbiology.

We thank Referee 2 for their positive comments and support for the work presented in this manuscript.

Referee #3 (Remarks to the Author):

I have commented on all aspects (key results, originality, data, statistics, etc) in my original review; besides, some addressal of text revisions I cannot see additional analyses or any deep reflection on my comments from the first round of reviews. The authors do state "we focused the manuscript on the construction of the GBR-MGD and associated technical advances. We agree with the reviewer that further investigation of observed community shifts will identify causal mechanisms (...). We are preparing a subsequent manuscript that focuses on the implicated taxa."

We have now included analyses of the microbial communities linked with water chemistry data to identify potential ecological drivers of community composition on the Great Barrier Reef (GBR) (lines 209-226). This analysis describes regional variation in community composition across the GBR and makes use of metagenomic data to highlight links between water chemistry and functional adaptations, such as an increase in phosphate transport genes to scavenge phosphate at reefs with low phosphate concentration.

In addition to the association of oligotrophic indicator microbes with no-take marine reserve (NTMR) zoning and copiotrophic indicators with fished reefs, we show that the enrichment of species is region specific and describe differences in functional gene signatures that suggest responses to distinct organic loads or nutrient profiles between regions (lines 269-310). These data support the notion that molecular data enables diagnostic reef monitoring that should be tailored to regions to increase accuracy and effectiveness. We have added a short commentary to link the insights gained from the GBR-MGD with improving reef monitoring on the GBR as suggested by Referee 2 (line 497-516).

Response to referees' comments

Referee #1 (Remarks to the Author):

The authors addressed all my points in the previous round of revision.

We thank Referee 1 for their time and feedback in reviewing the manuscript.
